# Supplementary material for: Co-design and evaluation of a youth-informed organisational tool to enhance trauma-informed practices in the UK public sector: a study protocol
Source: BMJ Open. 2024 Mar 12;14(3):e078545. doi: 10.1136/bmjopen-2023-078545 (PMC10936521; doi:10.1136/bmjopen-2023-078545)
Supplement: Author's manuscript [file bmjopen-2023-078545.draft_revisions.pdf]

BMJ Open

BMJ Open is committed to open peer review. As part of this commitment we make the peer review history of every article we publish publicly available.

When an article is published we post the peer reviewers' comments and the authors' responses online. We also post the versions of the paper that were used during peer review. These are the versions that the peer review comments apply to.

The versions of the paper that follow are the versions that were submitted during the peer review process. They are not the versions of record or the final published versions. They should not be cited or distributed as the published version of this manuscript.

BMJ Open is an open access journal and the full, final, typeset and author-corrected version of record of the manuscript is available on our site with no access controls, subscription charges or pay-per-view fees (http://bmjopen.bmj.com).

If you have any questions on BMJ Open's open peer review process please email info.bmjopen@bmj.com

BMJ Open

Co-design and evaluation of a youth-informed organisational tool to enhance trauma-informed practices in the UK public sector: a study protocol

Journal:	*BMJ Open*
Manuscript ID	bmjopen-2023-078545
Article Type:	Protocol
Date Submitted by the Author:	04-Aug-2023

Complete List of Authors:	Hugh-Jones, Siobhan ; University of Leeds Faculty of Biological Sciences, Psychology<br>Butcher, Isabelle; University of Oxford, Department of Psychiatry<br>Bhui , Kamaldeep; University of Oxford,

Keywords:	Child & adolescent psychiatry < PSYCHIATRY, MENTAL HEALTH, Health Education, Schools, PAEDIATRICS

SCHOLARONE™
Manuscripts

Co-design and evaluation of a youth-informed organisational tool to enhance trauma-informed practices in the UK public sector: a study protocol

Siobhan Hugh-Jones[1]* Isabelle Butcher[2] Kamaldeep Bhui[2]

[1] School of Psychology, University of Leeds, Leeds, LS2 9JT UK

[2] Department of Psychiatry, University of Oxford, Oxford, OX3 7JX UK

*Corresponding author s.hugh-jones@leeds.ac.uk

For peer review only

Abstract

Introduction A trauma-informed approach (TIA) means working with awareness that people's histories of trauma may shape the way the engage with a service, organisations/ institutions. Young people with adverse childhood experiences may be at risk of re-traumatisation by organisational practices. There are limited resources to help diverse public sector settings who work with adolescents to become trauma-informed, and needs of adolescents have not been central in their development. Gaps remain in the implementation and evaluation of resource to develop TIAs in the public sector. This study contributes to public sector capacity to work in trauma-informed ways with adolescents by co-designing and evaluating the implementation of a youth-informed organisational resource.

Methods and analysis This is a modified Experience-based Co-design (EBCD) study followed by pre-post evaluation. Public sector organisations/services, and adolescents connected with them, will collaboratively reflect on arts-based lived experience data and national dataset analyses about the experience and impact of adverse childhood experiences and trauma on adolescents' mental health (Stage 1). This data will be drawn from a larger study. Collaboratively priorities for organisational responses will be identified (stage 2), a low-burden resource co-designed (stage 3), deployed for implementation in diverse settings (stage 4) and evaluation of that implementation (stage 5). We also explore cost-effectiveness considerations. The study will provide insights into what adolescents and

public sector organisations want from a TIA resource, the experience of services/ organisations in implementing this and recommendations for resource development and implementation.

Ethics and dissemination This is NHS HRA approved (23/WM/0105). Learning will be across study participants in a workshop at study- end. Knowledge products will include a website detailing the created resource and youth-created film documenting; the study process, resources, experiences of implementation. Dissemination will target the following settings but is not limited to these; academic, healthcare, and local government via knowledge-exchange events.

Strengths and Limitations (five)

- This study aims to aims to co-design, implement and evaluate a public health resource that will enable those working in public sectors to work in trauma informed ways.
- The information gathered in this study will provide a unique insight into the key aspects that should be included in a trauma informed resource.
- It is crucial that young people from a wide range of backgrounds, ages, and geographic locations have an opportunity to participate in this novel study. This study will endeavour to include young people and professionals through purposive sampling.
- This study will also investigate whether a co-designed resource is both acceptable and affordable.
- This study is recruiting within England and so it is acknowledged that the resource may not applicable to the devolved nations. It is hoped however that it will be applicable to these nations also.

Introduction

Adverse childhood experiences (ACEs) include events such as verbal, sexual, or physical abuse; neglect; parental separation or incarceration; substance misuse, domestic violence, or family mental illness, bullying, poverty, racism, death of significant others and multiple losses[1 2]. Children who have experienced ACEs are at elevated risk of poor mental health

For peer review only - http://bmjopen.bmj.com/site/about/guidelines.xhtml

outcomes compared to children who have not experienced one of these ACE[3]. Experiencing multiple ACEs can have particularly detrimental effects on a physical, mental, social and economic outcomes in childhood, adolescence and adulthood.[4 5]

Many ACEs meet the definition of trauma[6] as events that involve actual or threatened death, serious injury or sexual violence either by witnessing it or hearing about it.[7] Although many ACEs (e.g. homelessness) are not included in this definition of trauma, many ACEs have trauma effects [1]. In our work, ACEs are considered as potentially traumatising, especially in the absence of buffering, supportive relationships and contexts. Our focus is the long-term impact of ACEs on later adolescent mental health.

Trauma can powerfully shape a young person's psychobiology[8], influencing their mood, thoughts, emotions and behaviours in adolescence as well as how they view, make sense of and react to events and people (e.g. being on alert for harm from people, anticipating negative outcomes). Such responses are adaptive following trauma but can easily be misunderstood or misrepresented by others as unhelpful, disproportionate, confusing or defensive. Experiencing trauma places people at risk of re-traumatisation; this occurs when experiences that are mundane to others (e.g. meeting professionals, being asked to consent to a process) remind the person of their past trauma, and a trauma-based response is evoked (e.g. submission, disengagement, fear-based aggression)[9]. 18 Many community organisations and services, from schools to youth organisations and healthcare, routinely encounter young people who may have experienced ACEs but may be unaware of how the young person's trauma may be shaping their needs, engagement and behaviour with them[10 11].

Since the early 2000s, there has been increasing global attention to the ways that organisations, services, institutions and settings (hereafter organisations) could work in trauma-informed ways [12 13]. Trauma-informed approaches (TIAs), although still being defined, concur that the aim is not to treat trauma-related difficulties, which is the remit of trauma specific services. Rather, TIAs focus on people and processes, and seek to improve organisational understanding that trauma is often inflicted by someone previously trusted by a young person, and to appreciate the prevalence and impact of trauma on a person's neurological, biological, psychological and social development, as well as how surviving

For peer review only - http://bmjopen.bmj.com/site/about/guidelines.xhtml

adversity may be shaping a person's coping strategies[14].[15]. TIAs align with a social model of disability, which views societal lack of adaptation and flexibility to be generating disability rather it being an innate property of the person. The key principles of a TIA, which have broad international recognition, are safety, trustworthiness, choice, collaboration and empowerment[12 16]. Yatchmenoff et al. [14] summarise these as safety, empowerment, and self-worth.

In the UK, the government has produced guidance on TIAs for the health and social care sectors[17]. The guidance presents TIAs as understanding how the trauma-imprint can affect a person's ability to develop trusting relationships with organisations and staff alongside the importance of minimising the risk of re-traumatisation (which can undermine recovery) and of increasing a sense of control and autonomy for the trauma survivor.[12] The way organisations function, their policies and procedures, and how they engage with and respond to people can all carry re-traumatisation risks[18]. The guidance also acknowledges that trauma, both direct and vicarious, can affect staff and thus protecting their wellbeing is also a feature of a TIA[16].

TIAs are widely recommended, and often implemented, across public sectors in the U.K [16 19 20] including healthcare, education and social services. Although the evidence of effectiveness is only emergent, and is highly complex to gather, TIAs appear to have potential to be beneficial for ACE-affected populations[21 22 23]. However, despite an organisation's commitment to ideology of a TIA, they can, like any complex intervention, be hard to implement and sustain [24 25]. They require change at a systemic level to support paradigm shifts across policy, procedures and practices, finding ways to in trauma-informed ways in every interaction and to prioritise the building of trusting, mutual relationships[26]. Organisations need support to become TI.

Multiple resources and toolkits have been developed internationally to assist organisations in implementing a TIA[27 28]. Toolkits have begun to emerge in the UK. For example, the Roots framework[26] is a self-assessment tool facilitating organisational reflection on current and possible practice, including in National Health Service settings. The Scottish government have produced a Trauma-Informed Practice Toolkit for sector-wide organisations to self-evaluate and plan action across ten implementation domains[29]. However, despite a

For peer review only - http://bmjopen.bmj.com/site/about/guidelines.xhtml

proliferation of resources for TIAs, young people's perspectives in shaping these and for organisations serving young people, remain rare. There are no UK resources to support a move towards a TIA for organisations working specifically with adolescents. There is also a need for continued learning on successful implementation of TIAs, to see what TIA look like in practice in different human services organisations, the barriers encountered and how these can be overcome[14] .A further significant gap in knowledge is the impact of TIAs. Existing evidence comes mostly from clinical settings, where professionals are likely to have specialist trauma training. There has been little evaluation of TIAs in UK public sector organisations whose primary focus is not clinical care.

Our *Attuned to Trauma* study aims to contribute to filling these knowledge gaps. We present our protocol for this study which is nested within a large, UK project (*Attune*) examining ACEs and pathways to mental health outcomes in adolescents. Our nested study builds on the learning from two early work streams in Attune, spanning arts-based lived experience and data set analyses. Combining insights from those work streams and using experience-based co-design (EBCD) principles[30], we bring young people and public sector organisations together to co-design, implement and evaluate a new youth-informed resource to support TIA in organisations relevant to adolescents (10-24y) in the UK. We commit to keeping youth voice central in this study to address gaps in the use of lived experience in the developing of TIAs.

Study Aim

This study aims to co-design, implement and evaluate a public health resource to help diverse public sector organisations in the UK to work in TI ways with adolescents and young people (10 - 24y). Our study also aims to contribute to understanding about how to deliver co-production studies with young people in ways that are trauma-sensitive.

Research questions

We address two methodological and three substantive research questions:

1. What do ACE-affected young people want to see in a trauma-sensitive, co-production approach? (methodological)

For peer review only - http://bmjopen.bmj.com/site/about/guidelines.xhtml

2. How does new knowledge (arts-based, experiential and statistical) about the effects of ACEs and trauma on adolescents impact UK public sector organisations who serve young people? (methodological)
3. What do young people and stakeholders want in a co-designed UK resource to improve public sector capacity to work in trauma-informed ways? (substantive0
4. Is such a co-designed resource acceptable, feasible, efficacious and affordable? (substantive)
5. How can the resource be refined for future implementation in the UK public sector? (substantive)

2.0 Methods

2.2 Design

This is a co-design and pre-post evaluation study nested within the UK Attune project. The co-design stage draws on EBCD which is widely used to design health service improvements. Central to EBCD is an initial discovery phase, which brings together services users and service providers in a balanced power relationship to discuss the personal experiences of service users and key moments in the service that shaped this. Services users and providers are then supported to co-design service changes to improve the quality of care[31]. Adapting EBCD to local projects and organisations is common. Our study adapts EBCD by using, in the discovery phase, (i) using early Attune insights from adolescents, instead of the usual service user interviews, and (ii) adolescent-produced art (which includes film) and narratives to convey lived experience instead of a purposefully commissioned service user 'trigger' film. We retain the core principle of EBCD in that the experience of young people will drive co-design of organisational resource for a TIA. The produced resource will be shared with specific organisations to explore how they chose to implement it. We will conduct a preliminary evaluations of this alongside exploratory cost-effectiveness analysis.

Normalisation Process Theory (NPT) and principles of diffusion of innovation[32] in service organisations will inform our approach to resource co-production, trial implementation and evaluation, acknowledging we are at the earliest stage of innovation for service development. NPT characterises implementation as a social process of collective action. It

For peer review only - http://bmjopen.bmj.com/site/about/guidelines.xhtml

proposes concentration on four domains at all stages of complex intervention design, delivery and evaluation, namely; intervention coherence, cognitive participation (engagement), collective action (required to enable the intervention to happen) and reflexive monitoring (of the costs and benefits of the innovation). Attention to these domains will structure our project approach. We will supplement these with the most relevant domains influencing the diffusion of innovations in services and organisations, e.g. characteristics of the system, the innovation and anticipated individual adopters [32 33]

2.3 Setting

This is a multi-site study taking place across three regions of England, namely Falmouth, Kent and Leeds, recruiting from their wider areas of London, Oxford, Yorkshire and Cornwall. These sites are chosen to support participation by young people and stakeholders from rural, coastal and urban regions. Place is important in understanding ACEs [34 35 36] and in understanding local organisational cultures and resources for change.

2.4 Participants

2.4.1 EBCD stage

There is little literature to inform trauma-informed, co-design methods (see [37 38]for example in primary healthcare intervention co-design), although principles of co-design overlap with some principles of a TIA (e.g. collaboration, choice). We aim to contribute to emerging discussions about how to deliver trauma-informed co-production methods by recruiting five young people from the Attune regional young people's advisory groups (YPAGs) to form a working party to design the EBCD workshops to be trauma-sensitive. The YPAGs are based in several regions in the UK: Cornwall, Kent, London, Oxfordshire and Yorkshire. The YPAGs comprise young people between 10-24y with diverse identities (e.g. in terms of neurodivergence, gender identity, ethnicity and gender).

For the EBCD workshops themselves, we will recruit approximately 15 young people (10-24y) and 15 professionals to attend workshops in each of our UK regions (Leeds, Falmouth, Kent; total n = 90). Young people will be recruited from groups already involved in the Attune project, supplemented with local recruitment via networks as needed. Purposive sampling of young people will be used to ensure diversity with regards to age, ethnicity, gender and place, and as far as possible, we will recruit young people who have experience

For peer review only - http://bmjopen.bmj.com/site/about/guidelines.xhtml

For peer review only

of the sector or organisation represented by professionals attending the workshops. To be eligible, a young person must: be between 10-24y; be able to give consent and, if under 16, provide guardian consent; have experienced trauma and / or ACEs; and be able to contribute to EBCD workshops (conducted in English) where ACEs and trauma will be discussed. All young people will be paid for their time in line with national guidance[39].

Professionals will be recruited from diverse public sector organisations in our nominated regions. We will seek organisations who have a helping role with young people, for example schools, local authorities, colleges and universities, youth offending teams, community support organisations, housing associations etc. Bringing diverse organisations together to work on TIAs can be effective when they are supported to focus on their similarities, common challenges and collective solutions rather than differences[14]. Working in local, regional contexts can support interprofessional learning[40]. To be eligible, organisations must confirm they are ready and motivated towards TI ways of working and commit to sending up to two organisational members of staff to all EBCD workshops.

2.4.2 Implementation and Evaluation

Between 10-15 organisations across our study regions will be involved in implementing and evaluating the co-designed resource for 6 months. If demand for implementation is higher than this, we will purposively select organisations to represent diverse settings and places. Organisations will be eligible to take part in this stage if they were involved in EBCD and report organisational readiness to implement the resource and support its evaluation by the Attune project. Assessing system readiness can be complex [40]we will use relevant aspects of the NASSS framework which considers influences, including system readiness, on the Non-adoption, Abandonment, Spread, Scale-up and Sustainability of patient-facing health and care technologies [33].

2.5 Procedure

2.5.1 EBCD stage

Pre-Workshop Preparation (Summer 2023)

Taking part in co-production for TIAs may generate individual and organisational concerns, e.g. around one's own trauma, managing personal and organisational disclosures, or about organisational limits for change. As part of our efforts to design trauma-informed co-

For peer review only - http://bmjopen.bmj.com/site/about/guidelines.xhtml

production methods, we will prioritise the fostering of psychological safety, a first principle of a TIA, for organisation staff. Applied to working practices, psychological safety is seen as essential to 'unfreezing' organisations so they can learn and change [41] and is manifested by "the willing contribution of ideas and actions to a shared enterprise" ([42] p. 24). It can be fostered by reducing perceived threats and interpersonal risk and encouraging provisional tries towards change [43]. Therefore, using a topic guide informed by NPT and Greenhalgh et al.'s [32 33] work on developing and implementing complex interventions, we will conduct approximately three individual online pre-workshop orientation meetings with organisations to help us understand their reasons for taking part, their needs and expectations of the study, the main demographic of young people they encounter, their current status with regards to TIAs, any strategic objectives informing their current or planned organisational delivery or organisational practice, their perceived risks around participation and how we can mitigate these (i.e. system antecedents and readiness for innovation).

We will also endeavour to promote psychological safety for adolescent attendees. For adolescents, psychological safety can means feeling able to take interpersonal risks because there is little fear that this will result in embarrassment, ridicule, or shame and so it enables people to engage, connect, change, and learn[42]. Positive relationships, allyship through working with peers similar to you, and control over contributions can all build psychologically safe working contexts for adolescents[44]. Towards this, we will conduct online engagement meetings with adolescents to begin to build positive relationships, to understand their motivations, needs, expectations and concerns about participation, and how we can mitigate any perceived risk, and optimise their ability to contribute. Adolescent attendees will also be invited to meet each other online before the first workshop and to identify shared ambitions for their involvement.

With consent, and with the help of the youth working party, appropriate information from the online pre-meetings with organisations and young people (e.g. strategic objectives of the organisation, perspectives on how to contribute) will be synthesised into a collaborator's booklet so that organisations and young people are aware in advance of who they will be working with in the workshops and how people can contribute and benefit differently.

For peer review only - http://bmjopen.bmj.com/site/about/guidelines.xhtml

Signed informed consent for the ECBD workshops will be secured after these pre-meetings from all confirmed attendees. Our learning from these meetings will shape workshop design. We will also secure opt-in or opt-out preference for photography and filming in the workshops.

EBCD Workshop Series

We will conduct four full day EBCD workshops over 18 months; three before the evaluation stage and one after for optimisation after learning lessons from the evaluation. Table 1 shows the aims and anticipated outcomes of each workshop and Figure 1 shows the study timeline and connection with the main Attune project. Young people will lead many workshop sessions and workshop facilitators will be members of the Attune research team who will be trained and follow a standardised format for each workshop.

(FIGURE 1 the timeline of the study and connection to wider Attune Project)

Workshops will involve activities and discussion in small and large groups, and we will endeavour for organisations to be paired with at least two young people (familiar to them) who use their organisation or who spend time in their setting. This will be arranged collaboratively. Discussion questions will be shaped by the youth working party so they are framed in ways that are meaningful and accessible to adolescent attendees; as far as possible, planned questions and activities will be sent to all attendees in advance of workshops. We will emphasise our valuing of multiple ways of contributing beyond talking (including listening, responding to anonymised digitised questions such as Mentimeter©, post-event feedback) from which we can learn. These are features of psychologically safe ways of working with young people. We will seek brief workshop evaluation via email after each workshop.

Data collection

EBCD workshops will be audio-recorded (small group and whole group level) and field notes will be taken by trained facilitators. We will collect all workshop artefacts (e.g. creative

For peer review only - http://bmjopen.bmj.com/site/about/guidelines.xhtml

outputs, written and digital responses) to support analysis. As soon as possible after each workshop, facilitators will meet to share notes and to reach consensus on key insights from the events. A live animator will attend all workshops to capture key messages to support post-workshop communications to attendees and the Attune project. At the end of the EBCD stage, we will evaluate our approach using the Public and Patient Engagement Evaluation Tool which assesses the quality and impact of public and patient engagement activities within health system organisations[45].

Data analysis

Audio-recordings of workshop discussions will be transcribed and linked only to organisation type where relevant. Otherwise, full anonymisation of any identifying details will occur at the point of transcription. Data analysis will focus on transcribed audio-recordings, supplemented by artefacts, using Framework Analysis [46]. Comparing and contrasting data is integral to Framework Analysis and it structures the examination of data within and across cases, which could be individuals, organisations and / or regions in our study (Gale et al., 2013). Analysis will be conducted at regional levels before being merged for whole group analysis. The larger Attune study includes multi-disciplinary expertise in this method. Data storage and sharing will be in line with the data management plan for the Attune project.

Workshop 1 (Autumn 2023)

The EBCD discovery phase typically involves understanding the organisation and 'service users' lived experiences. Workshop 1 is a discovery phase where we share Attune Workstream 1 data on the lived experiences of ACE-affected young people, generated using arts-based approaches, their views on how this has affected their mental health and how the insights are relevant for organisations. We will also present insights from Attune Workstream 2 which involves the analysis of several existing, large UK datasets to identify determinants of adolescent mental health risk and resilience following ACEs. Discussion will then explore key touchpoints [47]namely (i) how these insights about the imprint of trauma on adolescents lead organisations to reflect on and identify some prominent current practices which may be unattuned to this knowledge; and (ii) what kinds of help organisations feel would help them to become more youth- and trauma-informed. We avoid introducing

For peer review only - http://bmjopen.bmj.com/site/about/guidelines.xhtml

existing tools at this point to ensure service and service-user led solution. ATTUNE is including an economic component and we will seek attendee's views on the main economic consequences (e.g. increased use of certain services) of ACEs they are involved in addressing.

Workshop 1 ⟶ Workshop 2

In line with our data analysis plan, we will examine how the discovery phase impacted different sectors / organisations, and where the identified priorities and opportunities for change. Our analysis will be attentive to organisation type. Outcomes will be summarised by organisation type or sector and region first and then combined with outcomes from the other study regions, where appropriate. Regional and merged outcomes will be shared with regional attendees who will be encouraged to share and discuss this in their organisation.

Workshop 2 (Spring 2024)

As stimuli for Workshop 2, and if Workshop 1 data indicates relevance, we will share in advance with participants two existing UK toolkits for organisations to become more trauma-informed[26]. These toolkits are recent and evolving but are not yet youth informed and are resource-intensive to implement. In Workshop 2, we will begin co-design of a low-intensity, youth informed organisational resource. The aims of this workshop are to (i) identify modifiable ways of working to become trauma-informed and which are highly likely to be of benefit to adolescents; (ii) likely to be adopted (as per Greenhalgh et al., 2004 characteristics of the innovation and system); and (iii) develop the nature of resource to be created. We will also seek to identify the financial requirements for developing and utilising the resources. If consensus from co-design is lacking across our regional sites, we will convene a small working group with representation from each site to produce a final set of priorities [48] . These will especially focus on specifications that permit the resources to be adopted for particular sectors, regions, and intersectional characteristics encountered in stakeholder organisations.

Workshop 2 ⟶ Workshop 3

Once outputs are analysed, the research team and youth working party will create up to four outline prototype resources that satisfy some of the needs expressed in the workshops.

For peer review only - http://bmjopen.bmj.com/site/about/guidelines.xhtml

We will also produce draft implementation and evaluation recommendations, all shared in advance of Workshop 2, so that organisations have opportunities to discuss at a local level.

Workshop 3 (Easter 2024)

The aim of this workshop is to select and build the core components of a final resource, as well as final recommendations for its implementation and evaluation. Workshop outputs will inform development, with the youth working party, of a final resource ready for the implementation and evaluation phase. The impact of the resource on the likely use of health and social care services as well as other economic impacts will be discussed.

Workshop 4 (Spring 2025)

After the implementation and evaluation phase, Workshop 4 will bring participants back together to (i) share group-level analysis of outcomes and experience of implementation and (ii) key aspects of the toolkit to refine for testing in future studies. We will capture organisational plans to continue with any TIA changes, as well as youth and professional evaluations of study participation, including psychological safety.

For peer review only

Table 1: Aims and anticipated outcomes from EBCD workshops and informing concepts

Workshop	Primary Aim	Secondary Aim	Outcomes	Informing Concepts
**1**	To share lived experience and data insights from and about young people's ACEs and trauma (from Attune) to prompt reflection on organisational change.	Establish collaboration, model a trauma-informed way of working, promote youth voice, understand the readiness and needs of organisations from youth and professional perspectives to act on new knowledge.	Learning what the data means to diverse sectors and where organisations recognise need, motivation and opportunity to operationalise a TIA.	Linkage*<br>Communication & Influence*<br>Absorptive capacity for new knowledge*<br>System readiness*
**After Workshop 1:** synthesise outcomes across regions ready for sharing at Workshop 2.<br>If appropriate, share existing organisational TI principles and toolkits as stimulus.				
**2**	To identify priority, modifiable ways of working to become trauma-informed and identify the nature of a resource to be created.	To strengthen partnerships, to understand where youth and organisational perspectives on the nature of a TIA align and where they do not; and to understand the values and motivations of organisations for change, and what they need from a resource to help them do this, some expected outcomes for evaluation	Learning what organisations need from a resource, the most modifiable aspects of their ways of working, and likely benefit to them and young people.	Linkage*<br>The innovation*<br>Adopter*<br>System readiness*
**After Workshop 2:** synthesise outcomes and share.<br>Produce toolkit prototypes, and draft implementation and evaluation plans				
**3**	To select and refine core components of the final resource prototype	Decide on implementation options for the next phase and establish priorities for the evaluation.	Prototype resource and recommendations for implementation and evaluation.	Linkage*<br>Implementation Process*
**After Workshop 3:** Refine prototype resource, implementation plan and evaluation protocol.				
**4**	To review findings from the implementation and evaluation phase and	To capture implementation experiences and recommendations for an improved implementation protocol; to	Understanding how to improve the resource, and its implementation	NPT domains of evaluation: coherence, cognitive participation,

	improvements needed to the resource	understand lessons learned and ambitions of youth and organisation for TIA in the future	and evaluation, ready for future testing.	collective action, reflexive monitoring**

* From Greenhalgh et al (2004) conceptual model of the determinants of diffusion, dissemination and implementation of innovations in health service delivery and organisation.

**NPT (Murray et al., 2014)

For peer review only

2.5.2 Implementation and Evaluation

Implementation of new innovations can be optimised by the involvement stakeholders in the earliest stages of design and ownership over local and flexible implementation[32 49]. Organisations involved in EBCD stage will be invited to implement the resource in their setting for a minimum of six months. Although EBCD will produce implementation recommendations, organisations will have autonomy in adapting the resource and its implementation to their setting, reflecting a supported 'let it happen' approach to learn from organisational agency and creativity in implementation[50]. This is in line with moves away from prioritising intervention fidelity over local tailoring, especially in the learning phase of interventions [51].As a minimum, and in line with recommendations for TIA, organisations will be encouraged to establish an implementation team to lead and champion resource implementation in their setting [14].

Evaluation of the impact of TIA on service users is extremely complex [23]and is not the focus of our early-stage evaluation. We will evaluate resource implementation. Administering a standardised pre-post measure of an organisation's TIA may not be feasible as there are no UK generated measures suitable for evaluation of TIA in diverse settings such as those likely to be involved in our study. Thirkle et al.'s review [52]of measures of TIAs shows that most tools originated in the United States and are not easily tailorable beyond healthcare organisations[12].Decisions on whether to utilise a standardised measure or to generate a bespoke pre-post evaluation tool will be make in the EBCD stage. Evaluation will be informed by NPT and relevant domains of diffusion of innovations in services [32 33 49].It will include, as a minimum, a survey of how organisations deployed the resource and audio-recorded, key informant semi-structured interviews, following a topic guide, to explore: the experiences of organisations; barriers and solutions to resource use; any unanticipated benefits or adverse impacts; lessons learned and recommendation for improvements, testing and upscaling. Impacts on the local health and social care economies will be investigated. We will also consider the extent to which NPT parameter for optimal implementation were met: (coherence, cognitive participation, collective action and reflexive monitoring). As in all intervention research, it is important to learn if outcomes, especially poor ones, were influenced by the resource itself or by challenges in

For peer review only - http://bmjopen.bmj.com/site/about/guidelines.xhtml

implementation [53]. Qualitative data will be analysed using Framework Analysis, which is suitable for mixed methods data in health research[46 54].

3.0 Ethics and Dissemination

This study has received ethical approval from the UK National Health Service Health Research Authority (23/WM/0105 June 2023). Recruitment for the project will commence in summer 2023 and all study activity will be completed by August 2025. Organisations and participants will receive age-appropriate verbal and written information about the study, including as much transparency as possible about the workshops, who will be there, what is involved and how we are endeavouring to make the workshops psychologically safe. Participants will be reminded that they are free to take breaks in the workshop, to contribute in ways that are right for them, including just listening. Support for participants will be available in the workshops from trained clinicians and options for support beyond this will be made clear. Organisational consent for the implementation phase will be secured after the EBCD stage. For any level of study participation, we will secure signed informed consent, with additional guardian consent for under 16s, and opt-out consent for the filmmaking components. All participating adolescents and organisations, and their data, will be fully anonymised in any study outputs.

Dissemination will be within this study first, via Workshop 4 in the EBCD series to the study participants and organisations, and then to the larger Attune study. Wider knowledge dissemination will be in partnerships with adolescents and participating organisations, and will target academic, healthcare, education, social care, third sector and local government settings via knowledge exchange events, social media, executive reports, conference presentations and publications. We will share learning and insights about (i) conducting EBCD on trauma projects with adolescents and UK public sector settings, using arts-based approaches on lived experience; (ii) the concerns, priorities and opportunities adolescents and different sectors perceive for working in more trauma-informed ways; (iii) the resource they produced and how it was implemented in diverse settings; (iv) how resources for TIAs in diverse settings could be evaluated; and (v) next steps for resource refinement and upscaling, if appropriate. Knowledge products will include a study website detailing the

For peer review only - http://bmjopen.bmj.com/site/about/guidelines.xhtml

EBCD process, the resource, evaluation and outcomes, and a youth-created film documenting the study process and organisational experiences of implementation.

Funding:

This work was supported by the UKRI Medical Research Council. (MR/ W002183/1).

Patient and public involvement

Patients and/or the public were involved in the design, or conduct, or reporting, or dissemination plans of this research. Refer to the Methods section for further details.

Patient consent for publication

Not applicable

Competing interests:

None declared.

Acknowledgements

The authors wish to thank each member of the wider ATTUNE research team who have contributed to the wider work of the project.

Contributors

KB is the principal investigator for the ATTUNE study(ATTUNE — Department of Psychiatry (ox.ac.uk)) SHJ , KB and IB made substantial contributions to the conceptualisation and design of the study. IB and SHJ drafted the first version of the manuscript. IB, SHJ and KB reviewed the manuscript for intellectual content, approved the final version to be published and agreed to be accountable for all aspects of the work.

For peer review only - http://bmjopen.bmj.com/site/about/guidelines.xhtml

References

1. Varese F, Smeets F, Drukker M, et al. Childhood adversities increase the risk of psychosis: a meta-analysis of patient-control, prospective- and cross-sectional cohort studies. *Schizophr Bull* 2012;38(4):661-71. doi: 10.1093/schbul/sbs050 [published Online First: 2012/03/31]
2. Felitti VJMD, Facp, Anda RFMD, et al. Relationship of Childhood Abuse and Household Dysfunction to Many of the Leading Causes of Death in Adults: The Adverse Childhood Experiences (ACE) Study. *American Journal of Preventive Medicine* 1998;14(4):245-58. doi: 10.1016/S0749-3797(98)00017-8
3. Norman RE, Byambaa M, De R, et al. The long-term health consequences of child physical abuse, emotional abuse, and neglect: a systematic review and meta-analysis. *PLoS medicine* 2012;9(11):e1001349.
4. Hughes K, Ford K, Bellis MA, et al. Health and financial costs of adverse childhood experiences in 28 European countries: a systematic review and meta-analysis. *The Lancet Public Health* 2021;6(11):e848-e57.
5. Smith KE, Pollak SD. Early life stress and development: potential mechanisms for adverse outcomes. *Journal of neurodevelopmental disorders* 2020;12:1-15.
6. Friedman MJ, Resick PA, Bryant RA, et al. Classification of trauma and stressor-related disorders in DSM-5. *Depression and anxiety* 2011;28(9):737-49.
7. Association AP. Diagnostic and statistical manual of mental disorders (DSM-5®): American Psychiatric Pub 2013.
8. Ridout KK, Khan M, Ridout SJ. Adverse childhood experiences run deep: toxic early life stress, telomeres, and mitochondrial DNA copy number, the biological markers of cumulative stress. *Bioessays* 2018;40(9):1800077.
9. May CL, Wisco BE. Defining trauma: How level of exposure and proximity affect risk for posttraumatic stress disorder. *Psychological trauma: theory, research, practice, and policy* 2016;8(2):233.
10. Luthar SS, Mendes SH. Trauma-informed schools: Supporting educators as they support the children. *International Journal of School & Educational Psychology* 2020;8(2):147-57.
11. Spence R, Kagan L, Kljakovic M, et al. Understanding trauma in children and young people in the school setting. *Educational and Child Psychology* 2021;38(1):87-98.
12. Harris M, Fallot RD. Envisioning a trauma-informed service system: a vital paradigm shift. *New directions for mental health services* 2001;2001(89):3-22.
13. Maynard BR, Farina A, Dell NA, et al. Effects of trauma-informed approaches in schools: A systematic review. *Campbell Systematic Reviews* 2019;15(1-2)
14. Yatchmenoff DK, Sundborg SA, Davis MA. Implementing trauma-informed care: Recommendations on the process. *Advances in Social Work* 2017;18(1):167-85.
15. Fallot RD, Harris M. Creating cultures of trauma-informed care. *Washington DC: Community Connections* 2009
16. Fallot RD, Harris M. Trauma-informed approaches to systems of care. *Trauma Psychology Newsletter* 2008;3(1):6-7.
17. Office for Health IaD. Working definition of trauma-informed practice. 2022
18. Abuse S. SAMHSA's concept of trauma and guidance for a trauma-informed approach. 2014
19. Long E. The future of pastoral care in schools: exploring whole-school trauma-informed approaches. *Pastoral Care in Education* 2022;40(3):342-51.
20. Mendel WE, Sperlich M, Fava NM. "Is there anything else you would like me to know?": Applying a trauma-informed approach to the administration of the adverse childhood experiences questionnaire. *Journal of community psychology* 2021;49(5):1079-99.

For peer review only - http://bmjopen.bmj.com/site/about/guidelines.xhtml

21. Blanch A, Filson B, Penney D, et al. Engaging women in trauma-informed peer support: A guidebook. *Alexandria, VA: National Center for Trauma-Informed Care* 2012
22. Greenwald R, Siradas L, Schmitt TA, et al. Implementing trauma-informed treatment for youth in a residential facility: First-year outcomes. *Residential Treatment for Children & Youth* 2012;29(2):141-53.
23. Department for Levelling Up HaC. Trauma-informed approaches to supporting people experiencing multiple disadvantage 2023
24. Hodgdon HB, Kinniburgh K, Gabowitz D, et al. Development and implementation of trauma-informed programming in youth residential treatment centers using the ARC framework. *Journal of Family Violence* 2013;28:679-92.
25. Azeem MW, Aujla A, Rammerth M, et al. Effectiveness of six core strategies based on trauma informed care in reducing seclusions and restraints at a child and adolescent psychiatric hospital. *Journal of Child and Adolescent Psychiatric Nursing* 2011;24(1):11-15.
26. Thirkle SA, Kennedy A, Sice P, et al. The Co-Production of the Roots Framework: A Reflective Framework for Mapping the Implementation Journey of Trauma-Informed Care. *medRxiv* 2022:2022.04. 13.22273691.
27. Henderson CM, Everett, M. Isobel S (Sydney LHD). Trauma-Informed Care and Practice Organisational

Toolkit (TICPOT): An Organisational Change Resource, Stage 2 - Supporting Organisational Change. *Mental Health Coordinating Council (MHCC)*
28. Addington D, Addington J, Schissel B. A depression rating scale for schizophrenics. *Schizophrenia research* 1990;3(4):247-51.
29. Grandison AHaG. Trauma-Informed Practice: A toolkit for Scotland *Scottish Government*
30. The Point of Care Foundation : EBCD Experience based co design toolkit *Point of Care Foundation*
31. Moser A, Korstjens I. Series: Practical guidance to qualitative research. Part 5: Co-creative qualitative approaches for emerging themes in primary care research: Experience-based co-design, user-centred design and community-based participatory research. *European Journal of General Practice* 2022;28(1):1-12. doi: 10.1080/13814788.2021.2010700
32. Greenhalgh T, Robert G, Macfarlane F, et al. Diffusion of innovations in service organizations: systematic review and recommendations. *Milbank Q* 2004;82(4):581-629. doi: 10.1111/j.0887-378X.2004.00325.x [published Online First: 2004/12/15]
33. Greenhalgh T, Wherton J, Papoutsi C, et al. Beyond Adoption: A New Framework for Theorizing and Evaluating Nonadoption, Abandonment, and Challenges to the Scale-Up, Spread, and Sustainability of Health and Care Technologies. *J Med Internet Res* 2017;19(11):e367. doi: 10.2196/jmir.8775
34. Crouch E, Radcliff E, Probst JC, et al. Rural-urban differences in adverse childhood experiences across a national sample of children. *The Journal of Rural Health* 2020;36(1):55-64.
35. Wade Jr R, Cronholm PF, Fein JA, et al. Household and community-level adverse childhood experiences and adult health outcomes in a diverse urban population. *Child abuse & neglect* 2016;52:135-45.
36. Whiteside-Mansell L, McKelvey L, Saccente J, et al. Adverse childhood experiences of urban and rural preschool children in poverty. *International journal of environmental research and public health* 2019;16(14):2623.
37. McGeown H, Potter L, Stone T, et al. Trauma informed co-production: Collaborating and combining expertise to improve access to primary care with women with complex needs. *medRxiv* 2023:2023.04. 21.23288783.
38. Cherry L. Trauma Informed co-production Guidance *West Yorkshire Health and Care partnership*
39. NIHR. Payment guidance for researchers and professionals July 2022
40. Humphrey A, Eastwood L, Atkins H, et al. An exemplar of GP commissioning and child and adolescent mental health service partnership: Cambridge 1419 young people's service. *Journal of Integrated Care* 2016;24(1):26-37.

For peer review only

For peer review only - http://bmjopen.bmj.com/site/about/guidelines.xhtml

41. Schein EH, Bennis WG. Personal and organizational change through group methods: The laboratory approach: Wiley New York 1965.
42. Edmondson AC, Lei Z. Psychological safety: The history, renaissance, and future of an interpersonal construct. *Annu Rev Organ Psychol Organ Behav* 2014;1(1):23-43.
43. Frazier P, Richards D, Mooney J, et al. Acceptability and proof of concept of internet-delivered treatment for depression, anxiety, and stress in university students: protocol for an open feasibility trial. *Pilot and Feasibility Studies* 2016;2(1):28. doi: 10.1186/s40814-016-0068-9
44. Wanless SB. The Role of Psychological Safety in Human Development. *Research in Human Development* 2016;13(1):6-14. doi: 10.1080/15427609.2016.1141283
45. Abelson J, Li K, Wilson G, et al. Supporting quality public and patient engagement in health system organizations: development and usability testing of the P ublic and P atient E ngagement E valuation T ool. *Health Expectations* 2016;19(4):817-27.
46. Gale NK, Heath G, Cameron E, et al. Using the framework method for the analysis of qualitative data in multi-disciplinary health research. *BMC medical research methodology* 2013;13(1):1-8.
47. Donetto S, Tsianakas V, Robert G. Using Experience-based Co-design (EBCD) to improve the quality of healthcare: mapping where we are now and establishing future directions. *London: King's College London* 2014:5-7.
48. Robert G. Participatory action research: using experience-based co-design to improve the quality of healthcare services. *Understanding and Using Health Experiences–improving patient care* 2013:138-50.
49. Murray E, Treweek S, Pope C, et al. Normalisation process theory: a framework for developing, evaluating and implementing complex interventions. *BMC medicine* 2010;8:1-11.
50. May C. Towards a general theory of implementation. *Implementation Science* 2013;8(1):18. doi: 10.1186/1748-5908-8-18
51. von Thiele Schwarz U, Hasson H, Lindfors P. Applying a fidelity framework to understand adaptations in an occupational health intervention. *Work* 2015;51(2):195-203.
52. Thirkle S, Kennedy A, Sice P. Instruments for exploring trauma-informed care. *Journal of Health and Human Services Administration* 2021
53. Proctor E, Silmere H, Raghavan R, et al. Outcomes for implementation research: conceptual distinctions, measurement challenges, and research agenda. *Administration and policy in mental health and mental health services research* 2011;38:65-76.
54. Rashidian A, Eccles MP, Russell I. Falling on stony ground? A qualitative study of implementation of clinical guidelines' prescribing recommendations in primary care. *Health policy* 2008;85(2):148-61.

For peer review only

For peer review only - http://bmjopen.bmj.com/site/about/guidelines.xhtml

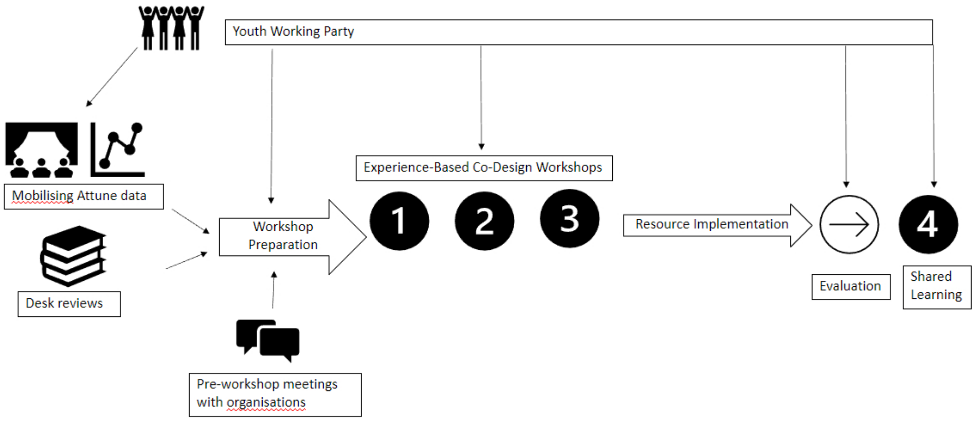

(FIGURE 1 the timeline of the study and connection to wider Attune Project)

307x157mm (96 x 96 DPI)

BMJ Open

Co-design and evaluation of a youth-informed organisational tool to enhance trauma-informed practices in the UK public sector: a study protocol

Journal:	*BMJ Open*
Manuscript ID	bmjopen-2023-078545.R1
Article Type:	Protocol
Date Submitted by the Author:	07-Dec-2023
Complete List of Authors:	Hugh-Jones, Siobhan ; University of Leeds Faculty of Biological Sciences, Psychology<br>Butcher, Isabelle; University of Oxford, Department of Psychiatry<br>Bhui , Kamaldeep; University of Oxford,
**Primary Subject Heading**:	Mental health
Secondary Subject Heading:	Paediatrics
Keywords:	Child & adolescent psychiatry < PSYCHIATRY, MENTAL HEALTH, Health Education, Schools, PAEDIATRICS

SCHOLARONE™
Manuscripts

Co-design and evaluation of a youth-informed organisational tool to enhance trauma-informed practices in the UK public sector: a study protocol

Siobhan Hugh-Jones[1]* Isabelle Butcher[2] Kamaldeep Bhui[2]

[1] School of Psychology, University of Leeds, Leeds, UK

[2] Department of Psychiatry, University of Oxford, Oxford, UK

***Corresponding author** s.hugh-jones@leeds.ac.uk Postal address: School of Psychology (Room 1.19) University of Leeds LS2 9JT Telephone number: T: ++44 113 343 5744

Keywords: trauma- informed, adolescent mental health, childhood adversity, Experienced- Based Co Design

Word Count: 5771 excluding title page, references, figures and tables

For peer review only - http://bmjopen.bmj.com/site/about/guidelines.xhtml

Abstract

Introduction A trauma-informed approach (TIA) means working with awareness that people's histories of trauma may shape the way the engage with a services, organisations or institutions. Young people with childhood adversities may be at risk of re-traumatisation by organisational practices in schools, universities, and by employers and health agencies when they seek support. There are limited evidence-based resources to help people working in diverse public sector to work with adolescents in trauma-informed ways and the needs of adolescents have not been central in their development. This study contributes to public sector capacity to work in trauma-informed ways with adolescents by co-designing and evaluating the implementation of a youth-informed organisational resource.

Methods and analysis This is an Accelerated Experience-based Co-design (EBCD) study followed by a pre-post evaluation. Public sector organisations or services, and adolescents connected with them, will collaboratively reflect on lived experience data assembled through creative arts practice, alongside data from epidemiological national datasets. These will present knowledge about the impact of adverse childhood experiences on adolescents' mental health (Stage 1). Collaboratively, priorities (touch points) for organisational responses will be identified (stage 2), and a low-burden resource will be co-designed (stage 3), and offered for implementation in diverse settings (stage 4) and evaluated (stage 5). This will provide insights into what adolescents and public sector organisations in the UK want from a TIA resource, the experience of organisations in implementing this and recommendations for resource development and implementation.

Ethics The UK National Health Service Health Research Authority approved this study (23/WM/0105). Knowledge products will include website detailing the created resource and a youth-created film documenting the study process, the elements of the co-designed resource, and experiences of implementation. Dissemination will target academic, healthcare, education, social care, third- sector, local government settings via knowledge exchange events, accessible briefings and publication.

Strengths and Limitations (five)

For peer review only - http://bmjopen.bmj.com/site/about/guidelines.xhtml

- This multi-site study nested study in a larger national study draws on youth lived experience and survey data to inform the co-design of a resource to support diverse public sector settings to work in trauma-informed ways.
- We plan to recruit diverse young people to work in partnership with the organisations through which they are recruited, so that youth voice informs the co-design process in all its aspects. Our study also aims to understand how to deliver co-production studies with young people in ways that are trauma-informed.
- Use of frameworks for experience-based co-design and implementation theory is a strength.
- Implementation plans and evaluation protocols will be co-designed with settings, improving the chances of acceptable and meaningful resource use and evaluation.
- A limitation is that the time for implementation is relatively short, and not formalised, relying on participants adopting the interventions in their work places; and evaluation stage is a simple real world pre-post assessment, not a trial.

For peer review only

Introduction

Adverse childhood experiences (ACEs) include events such as verbal, sexual, or physical abuse; neglect; parental separation or incarceration; substance misuse, domestic violence, or family mental illness, bullying, poverty, racism, death of significant others and multiple losses(1, 2). Children who have experienced ACEs are at elevated risk of poor mental health outcomes compared to children who have not experienced ACEs(3). Experiencing multiple ACEs can have particularly detrimental effects on a physical, mental, social and economic outcomes in childhood, adolescence and adulthood(4, 5).

Many ACEs meet the definition of trauma events(6) that involve actual or threatened death, serious injury or sexual violence, either by witnessing it or hearing about it(7). Although many ACEs (e.g. homelessness) are not included in this definition of trauma, many ACEs have trauma effects. In our work, ACEs are considered potentially traumatising, especially in the absence of buffering, supportive relationships and contexts(8). Our focus is the long-term impact of ACEs on later adolescent mental health.

For peer review only - http://bmjopen.bmj.com/site/about/guidelines.xhtml

Trauma can powerfully shape a young person's psychobiology(9), influencing their mood, thoughts, emotions and behaviours in adolescence as well as how they view, make sense of and react to events and people (e.g. being on alert for harm from people, anticipating negative outcomes). Such responses are adaptive following trauma, and dynamic in that responses to new traumas may change. However, usually the style of adaptation tends to persist even when a trauma has stopped. The response can overgeneralise into non-trauma situations(10) as threat perceptions are altered and sensitised.

Adaptive responses can include avoiding specific reminders, loud noises, too many people, or the need to calm and self-soothe at a time of anxiety and autonomic arousal linked to the flight-fight response. Alongside these there may be emotional dysregulation and impulsivity and an inability to plan if a person is affected by flashbacks and distress, as if the original event is being repeated. Therefore, a person's 'adaptive' behaviour and their responses can easily be misunderstood or misrepresented by others as unhelpful, disproportionate, confusing or defensive, or even showing poor conduct and unprofessional. This may result in actions by the teacher or employer, for example, to apply rules and codes of conduct, and sanctions.

These can then be felt as a form of re-traumatisation; this occurs when experiences that are mundane to others (e.g. meeting professionals, being asked to consent to a process) remind the person of their past trauma, and a trauma-based response is evoked (e.g. submission, disengagement, fear-based aggression(11)). Many community organisations and services, from schools to youth organisations and healthcare, routinely encounter young people who may have experienced ACEs but may be unaware of how the young person's trauma may be shaping their needs, engagement and behaviour with them(12, 13).

Since the early 2000s, there has been increasing global attention to the ways that organisations, services, institutions and settings (hereafter organisations) could work in trauma-informed ways(14, 15). Trauma-informed approaches (TIAs), although still being defined, concur that the aim is not to treat trauma-related difficulties, which is the remit of trauma specific services. Rather, TIAs focus on people and processes and seek to improve organisational understanding that trauma is often inflicted by someone previously trusted by a young person, and to appreciate the prevalence and impact of trauma on a person's

For peer review only - http://bmjopen.bmj.com/site/about/guidelines.xhtml

neurological, biological, psychological and social development, as well as how surviving adversity may be shaping a person's coping strategies(15, 16) . TIAs align with a social model of disability, which views societal lack of adaptation and flexibility to be generating disability rather it being an innate property of the person. The key principles of a TIA, which have broad international recognition, are safety, trustworthiness, choice, collaboration and empowerment (17, 18). Yatchmenoff et al.(15) summarise these as safety, empowerment, and self-worth.

In the UK, the government has produced guidance on TIAs for the health and social care sectors. The guidance presents TIAs as understanding how the trauma-imprint can affect a person's ability to develop trusting relationships with organisations and staff alongside the importance of minimising the risk of re-traumatisation (which can undermine recovery) and of increasing a sense of control and autonomy for the trauma survivor (17).The way organisations function, their policies and procedures, and how they engage with and respond to people can all carry re-traumatisation risks(19). The guidance also acknowledges that trauma, both direct and vicarious, can affect staff and thus protecting their wellbeing is also a feature of a TIA (18).

TIAs are widely recommended and often implemented across public sectors in the US, including healthcare, education and social services. Although the evidence of effectiveness is only emergent, and is highly complex to gather, TIAs appear to have potential to be beneficial for ACE-affected populations. However, despite an organisation's commitment to ideology of a TIA, they can, like any complex intervention, be hard to implement and sustain. They require change at a systemic level to support paradigm shifts across policy, procedures and practices, finding ways to in trauma-informed ways in every interaction and to prioritise the building of trusting, mutual relationships. Organisations need support to become TI.

Multiple resources and toolkits have been developed internationally to assist organisations in implementing a TIA. Toolkits have begun to emerge in the UK. For example, the Roots framework is a self-assessment tool facilitating organisational reflection on current and possible practice, including in National Health Service settings. The Scottish government have produced a Trauma-Informed Practice Toolkit for sector-wide organisations to self-

For peer review only - http://bmjopen.bmj.com/site/about/guidelines.xhtml

evaluate and plan action across ten implementation domains. However, despite a proliferation of resources for TIAs, young people's perspectives in shaping these remain rare(20). There is also a need for continued learning on successful implementation of TIAs, to see what TIAs look like in practice in different human services organisations, the barriers encountered and how these can be overcome. A further significant gap in knowledge is the impact of TIAs. Existing evidence comes mostly from clinical settings, where professionals are likely to have specialist trauma training. There has been little evaluation of TIAs in UK public sector organisations whose primary focus is not clinical care.

Our *Attuned to Trauma* study aims to contribute to filling these knowledge and practice gaps. We present our protocol for this study which is nested within a large, UK project (*Attune*) examining ACEs and pathways to mental health outcomes in adolescents. Our nested study builds on the learning from two early work streams in Attune, spanning arts-based lived experience and data set analyses. Combining insights from those work streams and using accelerated experience-based co-design (AEBCD) principles(21, 22), we bring young people and public sector organisations together to co-design, implement and evaluate a new youth-informed resource to support TIA in organisations relevant to adolescents (10-24y) in the UK. We utilise this age range as it is encouraged by the project funders (UK Research and Innovation) and reflects calls to appreciate the earlier onset and extension of this life phase, arguably driven by social, digital, marketing, education and economic forces(23). Working with young people, via participatory and co-design research methods, can increase the chances of service improvements which are sensitised to their needs and lived experience, and therefore more likely to be effective.(24) Our project aligns with co-production theory that 'service users' should not be seen as passive recipients of that service but as active citizens with something of value to contribute(25). Treating young people's perspectives as an equitable form of knowledge is a core theme of the UKRI funding stream for this project. We therefore bring professionals and young people into dialogue and co-creation together.

Study Aim

This study aims to co-design, implement and evaluate a public health resource to help diverse public sector organisations in the UK to work in TI ways with adolescents and young

For peer review only - http://bmjopen.bmj.com/site/about/guidelines.xhtml

people (10 - 24y). Our study also aims to contribute to understanding about how to deliver co-production studies with young people in ways that are trauma-sensitive.

Research questions

We address two primary research questions:

1. What do young people and stakeholders want in a co-designed UK resource to improve public sector capacity to work in trauma-informed ways?
2. Is such a co-designed resource acceptable, feasible, efficacious and affordable, and how should it be refined for future implementation in the UK public sector?

2.0 Methods

2.1 Patient and public involvement

Patients and/or the public were involved in the design, or conduct, or reporting, or dissemination plans of this research.

2.2 Design

This is a co-design and pre-post evaluation study nested within the UK Attune project. The co-design stage draws on EBCD which is widely used to design health service improvements. Central to EBCD is an initial discovery phase, which brings together services users and service providers in a balanced power relationship to discuss the personal experiences of service users and key moments in the service that shaped this. Services users and providers are then supported to co-design service changes to improve the quality of care. We will adopt an accelerated form of EBCD, which can be effective in achieving similar aims to standard EBCD(21, 22). AEBCD typically brings pre-existing narratives of experiences from relevant groups to start the co-design process, establishing where the co-design group identify consensus or divergence from those narratives. We will do this by bringing early Attune insights from adolescents, instead of the usual service user interviews, and adolescent-produced art (which includes film) and narratives to convey lived experience instead of a purposefully commissioned service user ‘trigger’ film. We retain the core principle of EBCD in that the experiences of young people will drive co-design of organisational resource(s) for a TIA. The produced resource(s) will be shared with specific organisations to explore how they chose to implement it. We will conduct a preliminary evaluation of this alongside an exploratory cost-effectiveness analysis.

For peer review only - http://bmjopen.bmj.com/site/about/guidelines.xhtml

Normalisation Process Theory (NPT) and principles of diffusion of innovation in service organisations will inform our approach to resource co-production, trial implementation and evaluation, acknowledging we are at the earliest stage of innovation for service development. NPT characterises implementation as a social process of collective action. It proposes concentration on four domains at all stages of complex intervention design, delivery and evaluation, namely; intervention coherence, cognitive participation (engagement), collective action (required to enable the intervention to happen) and reflexive monitoring (of the costs and benefits of the innovation). Attention to these domains will structure our project approach. We will supplement these with the most relevant domains influencing the diffusion of innovations in services and organisations, e.g., characteristics of the system, the innovation and anticipated individual adopters.

2.3 Setting

This is a multi-site study taking place across three regions of England, namely Cornwall, Kent and West Yorkshire. These sites are chosen to support participation by young people and stakeholders from rural, coastal and urban regions. Place is important in understanding ACEs and in understanding local organisational cultures and resources for change.

2.4 Participants

2.4.1 EBCD stage

There is little literature to inform trauma-informed, co-design methods (see for example in primary healthcare intervention co-design), although principles of co-design overlap with some principles of a TIA (e.g. collaboration, choice). We aim to contribute to emerging discussions about how to deliver trauma-informed co-production methods by recruiting five young people from the Attune regional young people's advisory groups (YPAGs) to form a working party to design the EBCD workshops to be trauma-sensitive. The YPAGs are based in several regions in the UK: Cornwall, Kent, London, Oxfordshire and Yorkshire. The YPAGs comprise young people between 10-24y with diverse identities (e.g. in terms of neurodivergence, gender identity, ethnicity and place).

Sample sizes for EBCD studies vary. The recommended number of services users is between 5-15(26, 27). For our AEBCD workshops, we aim to recruit 5-8 young people (10-24y) and between 5-8 professionals to attend workshops in each of our UK regions (Cornwall, West Yorkshire and Kent; highest total n = 48). Co-design processes need to be responsible to

For peer review only - http://bmjopen.bmj.com/site/about/guidelines.xhtml

participants with whom power is shared. Therefore, despite this intended plan, we will retain flexibility to respond to the needs of young people and stakeholders who may prefer sessions in smaller or larger groups or in a modified format.

Professionals will be recruited from diverse public sector organisations in our nominated regions. This may be through the Attune project network or by first approaches to local councils, local authorities, and / or third sector organisations. We will seek organisations who have a helping role with young people, e.g. schools, local authorities, colleges and universities, youth offending teams, community support organisations, housing associations etc. Bringing diverse organisations together to work on TIAs can be effective when they are supported to focus on their similarities, common challenges and collective solutions rather than differences (15).Working in local, regional contexts can support interprofessional learning. To be eligible, organisations must confirm they are ready and motivated towards TI ways of working and commit to sending up to two organisational members of staff to all EBCD workshops and to supporting the recruitment of two to three young people from their setting with whom they can work in partnership.

Young people will be recruited via their participating organisation / setting. Participating organisations will be asked to identify 2-3 young people from their setting who are: between 10-24y; able to give consent and, if under 16, provide guardian consent; have experienced trauma and / or ACEs; able to contribute to EBCD workshops (conducted in English) where ACEs and trauma will be discussed. Organisations will be asked to approach potential participants with study information and explore their interest in taking part and through to securing informed consent from them and their guardians as needed. In considering potential participants, organisations will be asked to consider inclusion of underrepresented groups where possible (i.e. young people from c minority communities, with diverse identities in terms of place, gender, ethnicity, LGBTG+ and neurodivergence). All young people will be paid for their time in line with national guidance (28). Having to secure guardian consent for under 16s, which is requirement of the approving ethics committee, may prohibit some young people from participating (e.g. those still living in adverse environments). Participation by professionals and young people is opt-in.

For peer review only - http://bmjopen.bmj.com/site/about/guidelines.xhtml

2.4.2 Implementation and Evaluation

All organisations who participated in the AEBCD across our study regions will be eligible for the next stage of implementing and evaluating the co-designed resource for 6 months.

2.5 Procedure

2.5.1 EBCD stage

Pre-Workshop Preparation (Summer 2023)

Taking part in co-production for TIAs may generate individual and organisational concerns, e.g. around one's own trauma, managing personal and organisational disclosures, or about organisational limits for change. As part of our efforts to design trauma-informed co-production methods, we will prioritise the fostering of psychological safety, a first principle of a TIA, for organisation staff. Applied to working practices, psychological safety is seen as essential to 'unfreezing' organisations so they can learn and change(29) and is manifested by "the willing contribution of ideas and actions to a shared enterprise" ((30)p. 24). It can be fostered by reducing perceived threats and interpersonal risk and encouraging provisional tries towards change(31). Therefore, using a topic guide informed by NPT and Greenhalgh et al.'s work(32, 33) on developing and implementing complex interventions, we will conduct approximately three individual online pre-workshop orientation meetings with organisations to help us understand their reasons for taking part, their needs and expectations of the study, the main demographic of young people they encounter, their current status with regards to TIAs, any strategic objectives informing their current or planned organisational delivery or organisational practice, their perceived risks around participation and how we can mitigate these (i.e. system antecedents and readiness for innovation).

We will also endeavour to promote psychological safety for adolescent attendees. For adolescents, psychological safety can means feeling able to take interpersonal risks because there is little fear that this will result in embarrassment, ridicule, or shame and so it enables people to engage, connect, change, and learn. Positive relationships, allyship through working with similar peers , and control over contributions can all build psychologically safe working contexts for adolescents(34). Towards this, we will conduct online engagement meetings with adolescents to begin to build positive relationships, to understand their motivations, needs, expectations and concerns about participation, and how we can

For peer review only - http://bmjopen.bmj.com/site/about/guidelines.xhtml

mitigate any perceived risk, and optimise their ability to contribute. Adolescent attendees will also be invited to meet each other online before the first workshop and to identify shared ambitions for their involvement.

With consent, and with the help of the youth working party, appropriate information from the online pre-meetings with organisations and young people (e.g. strategic objectives of the organisation, perspectives on how to contribute) will be synthesised into a collaborator's booklet so that organisations and young people are aware in advance of who they will be working with in the workshops and how people can contribute and benefit differently.

Signed informed consent for the AECBD workshops will be secured after these pre-meetings from all confirmed attendees. Our learning from these meetings will shape workshop design. We will also secure opt-in or opt-out preference for photography and filming in the workshops.

AEBCD Workshop Series

We will conduct four full day AEBCD workshops over 18 months; three before the evaluation stage and one after . Table 1 shows the aims and anticipated outcomes of each workshop and Figure 1 shows the study timeline and connection with the main Attune project. Young people will lead many workshop sessions and workshop facilitators will be members of the Attune research team who will be trained and follow a standardised format for each workshop.

(FIGURE 1 the timeline of the study and connection to wider Attune Project)

Workshops will involve activities and discussions in small and large groups, Discussion questions will be shaped by the youth working party so they are framed in ways that are meaningful and accessible to adolescent attendees; as far as possible, planned questions and activities will be sent to all attendees in advance of workshops. Conventions in AEBCD are to use basic but effective means of securing participants' engagement (e.g. creative

For peer review only - http://bmjopen.bmj.com/site/about/guidelines.xhtml

activities, scenarios, case discussion) and responses (e.g. flip charts, post-it notes, maps, Sketches etc). We offer examples below of the types of activities we may use and additional detail is given in Supplementary Materials. We will emphasise our valuing of multiple ways of contributing beyond talking (including listening, responding to anonymised digitised questions such as Mentimeter©, post-event feedback) from which we can learn. These are features of psychologically safe ways of working with young people. We will seek brief workshop evaluations via email after each workshop.

Data collection

AEBCD workshops will be audio-recorded (small group and/or whole group level) and field notes will be taken by trained facilitators. We will collect all workshop artefacts (e.g. creative outputs, written and digital responses) to support analysis. As soon as possible after each workshop, facilitators will meet to share notes and to reach consensus on key insights from the events. Data will be entered into excel spreadsheets were possible (e.g. listing specifics of artefacts) -At the end of the AEBCD stage, we will evaluate our approach using the Public and Patient Engagement Evaluation Tool which assesses the quality and impact of public and patient engagement activities within health system organisations(35).

Data analysis

The key moments in the workshop audio-recordings will be transcribed (i.e. those which are pertinent to learning, excluding for example, team building exercises). Transcribed extracts will only be linked to organisation type when essential, e.g. when knowledge of the setting type is required to comprehend the point / discussion. Full anonymisation of any other identifying details will occur at the point of transcription. Analysis will be conducted at regional levels before being merged for whole group analysis. We will merge the ‘key insights’ field notes with workshop artefacts and ‘key moments’ in the audio recording, initially framed in terms of workshop objectives (e.g. to understand views on an issue or to identify priorities). We will share emerging outcomes with the wider Attune project for credibility and sense-checking at key points. Lay summaries of each workshop will also be shared with participants prior to the next workshop, where they will be invited to approve

For peer review only - http://bmjopen.bmj.com/site/about/guidelines.xhtml

or improve our interpretation of learning from the preceding workshop. Data storage and sharing will be in line with the data management plan for the Attune project.

Workshop 1 (Autumn 2023)

The EBCD discovery phase typically involves understanding the organisation and 'service users' lived experiences. Workshop 1 is a discovery phase where we share Attune Workstream 1 data on the lived experiences of ACE-affected young people, generated using arts-based approaches, their views on how this has affected their mental health and how the insights are relevant for organisations. We will also present insights from Attune Workstream 2 which involves the analysis of several existing, large UK datasets to identify determinants of adolescent mental health risk and resilience following ACEs. Discussion will then explore key touchpoints(36) namely (i) how these insights about the imprint of trauma on adolescents lead organisations to reflect on and identify some prominent current practices which may be unattuned to this knowledge; and (ii) what kinds of help organisations feel would help them to become more youth- and trauma-informed. We avoid introducing existing tools at this point to ensure service and service-user led solution.

Workshop 1 → Workshop 2

In line with our data analysis plan, we will examine how the discovery phase impacted different sectors / organisations and where they identified priorities and opportunities for change. Our analysis will be attentive to organisation type if necessary (i.e. if differences emerge). Outcomes will be summarised by region first and then combined with outcomes from the other study regions, where appropriate. Regional and merged outcomes will be shared with regional attendees who will be encouraged to share and discuss this in their organisation.

Workshop 2 (Spring 2024)

As stimuli for Workshop 2, and if Workshop 1 data indicates relevance and need, we will share in advance with participants two existing UK toolkits for organisations to become more trauma-informed(37). These toolkits are recent and evolving but are not yet youth-informed and are resource-intensive to implement. In Workshop 2, we will begin co-design

For peer review only - http://bmjopen.bmj.com/site/about/guidelines.xhtml

of a low-intensity, youth-informed organisational resource. The aims of this workshop are to (i) identify modifiable ways of working to become trauma-informed and which are highly likely to be of benefit to adolescents; (ii) likely to be adopted (as per Greenhalgh et al., 2004 characteristics of the innovation and system(32)); and (iii) begin to develop the nature of resource to be created. If consensus from co-design is lacking across our regional sites, we will convene a small working group with representation from each site to produce a final set of priorities . These will especially focus on specifications that permit the resources to be adopted for particular sectors, regions, and intersectional characteristics encountered in stakeholder organisations.

Workshop 2 ⟶ Workshop 3

Once outputs are analysed, the research team and youth working party will create up to four outline prototype resources that satisfy some of the needs expressed in the workshops. We will also produce draft implementation and evaluation recommendations, all shared in advance of Workshop 2, so that organisations have opportunities to discuss at a local level.

Workshop 3 (Easter 2024)

The aim of this workshop is to select and build the core components of a final resource, as well as final recommendations for its implementation and evaluation. Workshop outputs will inform development, with the youth working party, of a final resource ready for the implementation and evaluation phase.

Workshop 4 (Spring 2025)

After the implementation and evaluation phase, Workshop 4 will bring participants back together to (i) share group-level analysis of outcomes and experience of implementation and (ii) key aspects of the resource to refine for testing in future studies. We will capture organisational plans to continue with any TIA changes, as well as youth and professional evaluations of study participation, including psychological safety.

For peer review only - http://bmjopen.bmj.com/site/about/guidelines.xhtml

Table 1: Aims and anticipated outcomes from EBCD workshops and informing concepts

Workshop	Primary Aim	Secondary Aim	Outcomes	Informing Concepts
**1**	To share lived experience and data insights from and about young people's ACEs and trauma (from Attune) to prompt reflection on organisational change.	Establish collaboration, model a trauma-informed way of working, promote youth voice, understand the readiness and needs of organisations from youth and professional perspectives to act on new knowledge.	Learning what the data means to diverse sectors and where organisations recognise need, motivation and opportunity to operationalise a TIA.	Linkage*<br>Communication & Influence*<br>Absorptive capacity for new knowledge*<br>System readiness*
**Met hods**	Presenting Attune artworks, films, key themes from Attune WS1 (lived experience extracts) and WS2 (outcomes of national dataset analyses). Enquiry activities about participants' views of this, the level of resonance / divergence, and what it	Pre-workshops communications and meetings to understand and build readiness and collaborative intention; being clear about the day's structure, content and purpose; dedicating safe spaces in the workshop and a trauma-informed facilitator to support young people; careful orientation (ice-breakers) opportunities;		

	directs attention to for public sector settings.	workshop activities based on play and arts (suitable for all ages) and multiple ways of contributing (drawing, writing, hand signals).		
**After Workshop 1:** synthesise outcomes across regions ready for sharing at Workshop 2. If appropriate, share existing organisational TI principles and toolkits as stimulus.				
**2**	To identify priority, modifiable ways of working to become trauma-informed and identify the nature of a resource to be created.	To strengthen partnerships, to understand where youth and organisational perspectives on the nature of a TIA align and where they do not; and to understand the values and motivations of organisations for change, and what they need from a resource to help them do this, some expected outcomes for evaluation	Learning what organisations need from a resource, the most modifiable aspects of their ways of working, and likely benefit to them and young people.	Linkage* The innovation* Adopter* System readiness*
**Methods**	Exploration of a select number of existing tools; imagination tasks for co-design; consensus-	Invigorating (through words) project ambition and potential through partnership; relationship building activities; activities exploring		

For peer review only - http://bmjopen.bmj.com/site/about/guidelines.xhtml

	reaching activities (nominal group technique; bullseye tasks); co-design activities	differences and similarities in adolescent and professionals' priorities; play building activities to explore opportunities and challenges in co-design		
**After Workshop 2:** synthesise outcomes and share. Produce toolkit prototypes, and draft implementation and evaluation plans				
**3**	To select and refine core components of the final resource prototype	Decide on implementation options for the next phase and establish priorities for the evaluation.	Prototype resource and recommendations for implementation and evaluation.	Linkage* Implementation Process*
**Met hods**	Goal agreement; Nominal group technique; priority rating activities for outcomes	Activities to explore aspects of NPT with professionals and (separately) to explore pro and cons of implementation options with young people; implementation roadmap design		
**After Workshop 3:** Refine prototype resource, implementation plan and evaluation protocol.				
**4**	To review findings from the implementation and	To capture implementation experiences and recommendations	Understanding how to improve the resource,	NPT domains of evaluation:

For peer review only - http://bmjopen.bmj.com/site/about/guidelines.xhtml

	evaluation phase and improvements needed to the resource	for an improved implementation protocol; to understand lessons learned and ambitions of youth and organisation for TIA in the future	and its implementation and evaluation, ready for future testing.	coherence, cognitive participation, collective action, reflexive monitoring**
**Methods**	Arts-based activities to capture experience; case discussions; comparative outcomes to expectations	Priority rankings for learning and development; creating key messages banners		

* From Greenhalgh et al (2004) conceptual model of the determinants of diffusion, dissemination and implementation of innovations in health service delivery and organisation.

**NPT (Murray et al., 2014)

For peer review only - http://bmjopen.bmj.com/site/about/guidelines.xhtml

2.5.2 Implementation and Evaluation

Implementation of new innovations can be optimised by the involvement stakeholders in the earliest stages of design and ownership over local and flexible implementation(32, 38). Organisations involved in the AEBCD stage will be invited to implement the resource in their setting for a minimum of six months. Although AEBCD will produce implementation recommendations, organisations will have autonomy in adapting the resource and its implementation to their setting, reflecting a supported 'let it happen' approach to learn from organisational agency and creativity in implementation. This is in line with moves away from prioritising intervention fidelity over local tailoring, especially in the learning phase of interventions(39). As a minimum, and in line with recommendations for TIA, organisations will be encouraged to establish an implementation team to lead and champion resource implementation in their setting(15) .

Evaluation of the impact of TIA on service users is extremely complex(40) and is not the focus of our early-stage evaluation. We will evaluate resource implementation. Administering a standardised pre-post measure of an organisation's TIA may not be feasible as there are no UK generated measures suitable for evaluation of TIA in diverse settings such as those likely to be involved in our study. Thirkle et al.'s review of measures(41)of TIAs shows that most tools originated in the United States and are not easily tailorable beyond healthcare organisations(17). Decisions on whether to utilise a standardised measure or to generate a bespoke pre-post evaluation tool will be make in the AEBCD stage. Evaluation will be informed by NPT and relevant domains of diffusion of innovations in services(32, 33, 38). It will include, as a minimum, a survey of how organisations deployed the resource and audio-recorded, key informant semi-structured interviews, following a topic guideto explore: the experiences of organisations; barriers and solutions to resource use; any unanticipated benefits or adverse impacts; lessons learned and recommendation for improvements, testing and upscaling._ We will also consider the extent to which the NPT parameters for optimal implementation were met:coherence, cognitive participation, collective action and reflexive monitoring). As in all intervention research, it is important to learn if outcomes, especially poor ones, were influenced by the resource itself or by challenges in implementation(42). Qualitative data will be analysed using Framework Analysis, which is suitable for mixed methods data in health research(43, 44).

For peer review only - http://bmjopen.bmj.com/site/about/guidelines.xhtml

3.0 Ethics and Dissemination

This study has received ethical approval from the UK National Health Service Health Research Authority (23/WM/0105 June 2023). Recruitment for the project will commence in summer 2023 and all study activity will be completed by August 2025. Organisations and participants will receive age-appropriate verbal and written information about the study, including as much transparency as possible about the workshops, who will be there, what is involved and how we are endeavouring to make the workshops psychologically safe. Participants will be reminded that they are free to take breaks in the workshop, to contribute in ways that are right for them, including just listening. Support for participants will be available in the workshops from trauma specialists and options for support beyond this will be made clear. Organisational consent for the implementation phase will be secured after the AEBCD stage. For any level of study participation, we will secure signed informed consent, with additional guardian consent for under 16s, and opt-out consent for the filmmaking components. All participating adolescents and organisations, and their data, will be fully anonymised in any study outputs.

Dissemination will be within this study first, via Workshop 4 in the AEBCD series to the study participants and organisations, and then to the larger Attune study. Wider knowledge dissemination will be in partnerships with adolescents and participating organisations, and will target academic, healthcare, education, social care, third sector and local government settings via knowledge exchange events, social media, executive reports, conference presentations and publications. We will share learning and insights about (i) conducting AEBCD on trauma projects with adolescents and UK public sector settings, using arts-based approaches on lived experience; (ii) the concerns, priorities and opportunities adolescents and different sectors perceive for working in more trauma-informed ways; (iii) the resource they produced and how it was implemented in diverse settings; (iv) how resources for TIAs in diverse settings could be evaluated; and (v) next steps for resource refinement and upscaling, if appropriate. Knowledge products will include a study website detailing the EBCD process, the resource, evaluation and outcomes, and a youth-created film documenting the study process and organisational experiences of implementation.

For peer review only - http://bmjopen.bmj.com/site/about/guidelines.xhtml

Discussion

Trauma is so widespread and its effects so significant that is it has been positioned as a significant public health issue.(45) ACEs and trauma are highly predictive of poor outcomes in all areas of life and across lifespans, with adolescence being a time of particular vulnerability to the presentation of poor mental health in ACE-affected young people. Trauma-informed ways of working with young people strive to understand the ways that their ACEs may be shaping their needs and engagement with a service, with efforts to minimise re-traumatisation. Although resources are emerging in the UK to support public sector settings to be trauma-informed, we are still at a preliminary stage of understanding about how they may be best implemented and sustained and whether they meet the needs of young people.

To our knowledge, this is the first AEBCD study working across different UK public sector settings to co-design a resource that could help professionals be more attuned to the needs of adolescents in their settings who have lived through ACEs and / or trauma. Our study turns into action the learning from the arts-based participatory methods in the main Attune study which captured the lived experience of diverse, ACE-affected adolescents in multiple UK regions. A number of strengths benefit the study. Involvement of different public sector settings allows us to identify commonalities in needs. We support collaboration between adolescents and professionals, inviting them to join the project together and work in dialogue across the ABCD workshops. We address the need to better understand how resources for TIA can be implemented and evaluated in the public sector, driven by settings / services themselves and informed by NPT(32). This study will produce new insights on what adolescents and professionals want in a co-designed UK resource to improve their capacity to work in trauma-informed ways, and whether that resource is feasible and helpful Providing resources for teachers and employers based on AEBCD means privileging lived experience to generate novel insights and interventions, positioning this as valid new data and not dismissing it as subjective experiences; doing so compounds epistemic injustice in research and in support. This work contributes to the national agenda for developing trauma-informed approaches for young people.(40)

For peer review only - http://bmjopen.bmj.com/site/about/guidelines.xhtml

The study has some limitations. Recruitment of young people under 16 will be subject to guardian consent; this may exclude young people who do not wish to or cannot secure that consent. The AEBCD process of three workshops may make project participation possible for stretched organisations who can only give limited time to research projects but means deep exploration and creativity may be compromised. Bringing diverse settings together may risk consensus-reaching on the type of resource to be created. The effects of trauma-informed organisational interventions can be slow to emerge and examine (40) Thus, in our time-limed project, our is an exploratory evaluation of their experiences implementing the resource to inform resource improvements, more effective implementation strategies and a more robust evaluation at a future point.

Funding:

This work was supported by the UKRI Medical Research Council. (MR/ W002183/1).

Patient consent for publication

Not applicable

Competing interests:

None declared.

Acknowledgements

The authors wish to thank each member of the wider ATTUNE research team who have contributed to the wider work of the project.

Contributors

KB is the principal investigator for the ATTUNE study(ATTUNE — Department of Psychiatry (ox.ac.uk)) SHJ , KB and IB made substantial contributions to the conceptualisation and design of the study. IB and SHJ drafted the first version of the manuscript. IB, SHJ and KB reviewed the manuscript for intellectual content, approved the final version to be published and agreed to be accountable for all aspects of the work.

For peer review only - http://bmjopen.bmj.com/site/about/guidelines.xhtml

For peer review only

References

1. Felitti VJ, Anda RF, Nordenberg D, Williamson DF, Spitz AM, Edwards V, Marks JS. Relationship of childhood abuse and household dysfunction to many of the leading causes of death in adults: The Adverse Childhood Experiences (ACE) Study. American journal of preventive medicine. 1998;14(4):245-58.
2. Varese F, Smeets F, Drukker M, Lieverse R, Lataster T, Viechtbauer W, et al. Childhood adversities increase the risk of psychosis: a meta-analysis of patient-control, prospective-and cross-sectional cohort studies. Schizophrenia bulletin. 2012;38(4):661-71.
3. Norman RE, Byambaa M, De R, Butchart A, Scott J, Vos T. The long-term health consequences of child physical abuse, emotional abuse, and neglect: a systematic review and meta-analysis. PLoS medicine. 2012;9(11):e1001349.
4. Hughes K, Ford K, Bellis MA, Glendinning F, Harrison E, Passmore J. Health and financial costs of adverse childhood experiences in 28 European countries: a systematic review and meta-analysis. The Lancet Public Health. 2021;6(11):e848-e57.
5. Smith KE, Pollak SD. Early life stress and development: potential mechanisms for adverse outcomes. Journal of neurodevelopmental disorders. 2020;12(1):1-15.
6. Friedman MJ, Resick PA, Bryant RA, Strain J, Horowitz M, Spiegel D. Classification of trauma and stressor-related disorders in DSM-5. Depression and anxiety. 2011;28(9):737-49.
7. Association AP. Diagnostic and statistical manual of mental disorders (DSM-5®) American Psychiatric Pub. 2013.
8. Von Cheong E, Sinnott C, Dahly D, Kearney PM. Adverse childhood experiences (ACEs) and later-life depression: perceived social support as a potential protective factor. BMJ open. 2017;7(9):e013228.
9. Ridout KK, Khan M, Ridout SJ. Adverse childhood experiences run deep: toxic early life stress, telomeres, and mitochondrial DNA copy number, the biological markers of cumulative stress. Bioessays. 2018;40(9):1800077.
10. Lis S, Thome J, Kleindienst N, Mueller-Engelmann M, Steil R, Priebe K, et al. Generalization of fear in post-traumatic stress disorder. Psychophysiology. 2020;57(1):e13422.
11. May CL, Wisco BE. Defining trauma: How level of exposure and proximity affect risk for posttraumatic stress disorder. Psychological trauma: theory, research, practice, and policy. 2016;8(2):233.
12. Luthar SS, Mendes SH. Trauma-informed schools: Supporting educators as they support the children. International Journal of School & Educational Psychology. 2020;8(2):147-57.
13. Spence R, Kagan L, Kljakovic M, Bifulco A. Understanding trauma in children and young people in the school setting. Educational and Child Psychology. 2021;38(1):87-98.
14. Maynard BR, Farina A, Dell NA, Kelly MS. Effects of trauma-informed approaches in schools: A systematic review. Campbell Systematic Reviews. 2019;15(1-2).
15. Yatchmenoff DK, Sundborg SA, Davis MA. Implementing trauma-informed care: Recommendations on the process. Advances in Social Work. 2017;18(1):167-85.

For peer review only - http://bmjopen.bmj.com/site/about/guidelines.xhtml

16. Fallot RD, Harris M. Creating cultures of trauma-informed care. Washington DC: Community Connections. 2009.
17. Harris M, Fallot RD. Envisioning a trauma-informed service system: A vital paradigm shift. New directions for mental health services. 2001;2001(89):3-22.
18. Fallot RD, Harris M. Trauma-informed approaches to systems of care. Trauma Psychology Newsletter. 2008;3(1):6-7.
19. Abuse S. Trauma-informed care. SAMHSA News. 2014;22(2).
20. Hickle K. Trauma Informed Evaluation Report. Hove: YMCA; 2018.
21. Locock L, Robert G, Boaz A, Vougioukalou S, Shuldham C, Fielden J, et al. Using a national archive of patient experience narratives to promote local patient-centered quality improvement: an ethnographic process evaluation of 'accelerated'experience-based co-design. Journal of Health Services Research & Policy. 2014;19(4):200-7.
22. Barker RE, Brighton LJ, Maddocks M, Nolan CM, Patel S, Walsh JA, et al. Integrating home-based exercise training with a hospital at home service for patients hospitalised with acute exacerbations of COPD: developing the model using accelerated experience-based co-design. International Journal of Chronic Obstructive Pulmonary Disease. 2021:1035-49.
23. Sawyer SM, Azzopardi PS, Wickremarathne D, Patton GC. The age of adolescence… and young adulthood–Authors' reply. The Lancet Child & Adolescent Health. 2018;2(4):e7.
24. Boyle D, Harris M. The challenge of co-production. London: new economics foundation. 2009;56:18.
25. Coyne I, Carter B. Participatory research in the past, present and future. Being participatory: Researching with children and young people: co-constructing knowledge using creative techniques. 2018:1-13.
26. Foundation PoC. EBCD: Experience-based co-design toolkit: Step by Step guide 2013 [Available from: https://www.pointofcarefoundation.org.uk/resource/experience-based-co-design-ebcd-toolkit/.
27. Centr TSC. The importance of participatory methods

to research and system change. 2019.
28. NIHR. Payment guidance for researchers and professionals July 2022.
29. Schein EH, Bennis WG. Personal and organizational change through group methods: The laboratory approach: Wiley New York; 1965.
30. Edmondson AC, Lei Z. Psychological safety: The history, renaissance, and future of an interpersonal construct. Annu Rev Organ Psychol Organ Behav. 2014;1(1):23-43.
31. Frazier P, Richards D, Mooney J, Hofmann SG, Beidel D, Palmieri PA, Bonner C. Acceptability and proof of concept of internet-delivered treatment for depression, anxiety, and stress in university students: protocol for an open feasibility trial. Pilot and feasibility studies. 2016;2(1):1-9.
32. Greenhalgh T, Robert G, Macfarlane F, Bate P, Kyriakidou O. Diffusion of innovations in service organizations: systematic review and recommendations. The milbank quarterly. 2004;82(4):581-629.
33. Greenhalgh T, Wherton J, Papoutsi C, Lynch J, Hughes G, Hinder S, et al. Beyond adoption: a new framework for theorizing and evaluating nonadoption, abandonment, and challenges to the scale-up, spread, and sustainability of health and care technologies. Journal of medical Internet research. 2017;19(11):e8775.
34. Wanless SB. The role of psychological safety in human development. Research in Human Development. 2016;13(1):6-14.

For peer review only - http://bmjopen.bmj.com/site/about/guidelines.xhtml

35. Abelson J, Li K, Wilson G, Shields K, Schneider C, Boesveld S. Supporting quality public and patient engagement in health system organizations: development and usability testing of the P ublic and P atient E ngagement E valuation T ool. Health Expectations. 2016;19(4):817-27.
36. Donetto S, Tsianakas V, Robert G. Using Experience-based Co-design (EBCD) to improve the quality of healthcare: mapping where we are now and establishing future directions. London: King’s College London. 2014:5-7.
37. Thirkle SA, Kennedy A, Sice P, Patel P. The Co-production of the Roots Framework: A Reflective Framework for Mapping the Implementation Journey of Trauma-informed Care. medRxiv. 2022:2022.04. 13.22273691.
38. Murray E, Treweek S, Pope C, MacFarlane A, Ballini L, Dowrick C, et al. Normalisation process theory: a framework for developing, evaluating and implementing complex interventions. BMC medicine. 2010;8:1-11.
39. von Thiele Schwarz U, Hasson H, Lindfors P. Applying a fidelity framework to understand adaptations in an occupational health intervention. Work. 2015;51(2):195-203.
40. HaC. DfLU. Trauma-informed approaches to supporting people experiencing multiple disadvantage. 2023.
41. Thirkle S, Kennedy A, Sice P. Instruments for exploring trauma-informed care. Journal of Health and Human Services Administration. 2021.
42. Proctor E, Silmere H, Raghavan R, Hovmand P, Aarons G, Bunger A, et al. Outcomes for implementation research: conceptual distinctions, measurement challenges, and research agenda. Administration and policy in mental health and mental health services research. 2011;38:65-76.
43. Gale NK, Heath G, Cameron E, Rashid S, Redwood S. Using the framework method for the analysis of qualitative data in multi-disciplinary health research. BMC medical research methodology. 2013;13(1):1-8.
44. Rashidian A, Eccles MP, Russell I. Falling on stony ground? A qualitative study of implementation of clinical guidelines’ prescribing recommendations in primary care. Health policy. 2008;85(2):148-61.
45. Magruder KM, McLaughlin KA, Elmore Borbon DL. Trauma is a public health issue. European journal of psychotraumatology. 2017;8(1):1375338.

For peer review only

For peer review only - http://bmjopen.bmj.com/site/about/guidelines.xhtml

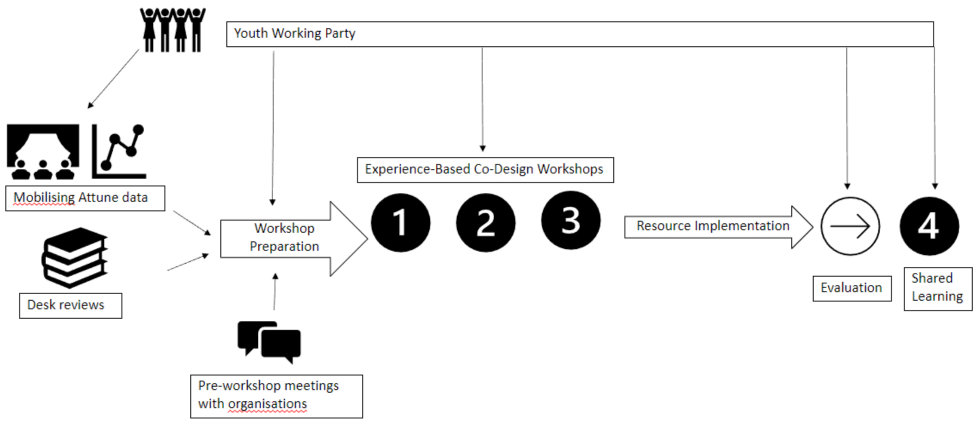

(FIGURE 1 the timeline of the study and connection to wider Attune Project)

307x157mm (96 x 96 DPI)

SUPPLEMENTARY MATERIALS

This table shows example activities that may be used in the AEBCD series in ways that can be tailored to different ages of young people, and which draw on creative practices and play.

	All ages	10-12 year olds	13-15 year olds	16+ year olds
Showing 'trigger films' (standard in EBCD)	Given context for films and purpose in workshop	Asked to spot 1-2 things in short clips (bingo). Given cards to show their view of important / not important / not sure in response to short clips.	Asked to write a word that captures what they felt was important in a clip. Collect and discuss as a group	Open discussion after clips. Encouraged to make brief notes during screening. Can use 'not sure' card
Sharing data / information	Presented in an engaging way; not using academic terms; giving examples; present as a quiz / myth busting / predicting what the information will say	Using thumbs up / down to indicate if they think it makes sense / is important / worth discussing.	Additionally asked for whether they are surprised by the data (thumbs up / down). Ideas for how we might explain the data / information to someone else.	In small groups, creating Venn diagrams / idea bubbles about what might they notice in the data / information and what might explain it
Persona / Character Play: designing a character with a back story to explore and better comprehend the nature of issues / diversity of experience	Given creative materials to design character in small group; asked to answer 'what has happened to your character'? 'how did that make them feel?' and 'what would they like to happen now'?	Supported to think about how movie characters are made up can still be about real life. Encouraged to focus on physical design of character first and then their story	Given guidance to think first about the big issue / experience they want their character to represent.	Encouraged to think about composite issues that a character could represent
Snowball Fight: people write an example on a piece of paper and in groups throw across the room for a set period. Pick up and read out examples near you at end.	Can write or draw the example, word they want to share.	Can ask someone else to read out word on the paper if wished.	Could be invited to think of the best word / example they can	Could be invited to add a brief statement word / example explaining why that is important to them
Forum Theatre: inviting or showing a live performance of a scenario, with pauses to ask the 'audience' different ways to respond / solve / change the scenario	Show example of how Forum Theatre works and how it can help creativity. Specific boundaries of response s as feasible and realistic in the real world.	Can be offered options if they find it difficult to imagine alternative responses and / or can focus ratings of big / medium / small problems in the scenarios	Supported to try one response option first; given time to consider impact and supported to report what they see.	Could hold opposing response options in mind and discussion in some detail
Bullseye Activity: refining the specific aims/ outcomes of a	Give examples of the pros and cons of specific vs.	Invited to design the bullseye page and work with same-age	Supported to think of 'wishes' or 'if only' as a	Supported to consider specific vs

For peer review only

resource or anticipated change	general aims, using everyday examples. Practising how we can vote for something as being near or far from our collectively determined bullseye	peers to generate ideas for outcomes, using comparisons to everyday examples	way to think about potential change / outcomes.	general aims via colour coding
Implementation mapping: decision trees / if-then steps	Shown examples of mapping resource implementation to understand choices and options, as well as importance of collective clarity	Use of simple ratings (Yes, No, Maybe) on cards for the most important choices. Moving these into piles to help see choices.	Use of 'must do', 'must not do', 'not sure' cards in response to options; adding of red and green flags for point of risk vs opportunity	Supported in groups to sketch starting point and key steps in implementation, considering what would be realistic in their setting.

For peer review only

BMJ Open

Co-design and evaluation of a youth-informed organisational tool to enhance trauma-informed practices in the UK public sector: a study protocol

Journal:	*BMJ Open*
Manuscript ID	bmjopen-2023-078545.R2
Article Type:	Protocol
Date Submitted by the Author:	08-Feb-2024
Complete List of Authors:	Hugh-Jones, Siobhan ; University of Leeds Faculty of Biological Sciences, Psychology<br>Butcher, Isabelle; University of Oxford, Department of Psychiatry<br>Bhui , Kamaldeep; University of Oxford,
**Primary Subject Heading**:	Mental health
Secondary Subject Heading:	Paediatrics
Keywords:	Child & adolescent psychiatry < PSYCHIATRY, MENTAL HEALTH, Health Education, Schools, PAEDIATRICS

SCHOLARONE™
Manuscripts

Co-design and evaluation of a youth-informed organisational tool to enhance trauma-informed practices in the UK public sector: a study protocol

Siobhan Hugh-Jones[1]* Isabelle Butcher[2] Kamaldeep Bhui[2]

[1] School of Psychology, University of Leeds, Leeds, UK

[2] Department of Psychiatry, University of Oxford, Oxford, UK

*Corresponding author s.hugh-jones@leeds.ac.uk

For peer review only

Abstract

Introduction A trauma-informed approach (TIA) means working with awareness that people's histories of trauma may shape the way the engage with a services, organisations or institutions. Young people with adverse childhood experiences may be at risk of re-traumatisation by organisational practices in schools and universities, and by employers and health agencies when they seek support. There are limited evidence-based resources to help people working in the public sector to work with adolescents in trauma-informed ways and the needs of adolescents have not been central in resource development. This study contributes to public sector capacity to work in trauma-informed ways with adolescents by co-designing and evaluating the implementation of a youth-informed organisational resource.

Methods and analysis This is an Accelerated Experience-based Co-design (AEBCD) study followed by pre-post evaluation. Public sector organisations or services, and adolescents connected with them, will collaboratively reflect on lived experience data assembled through creative arts practice, alongside data from epidemiological national datasets. These will present knowledge about the impact of adverse childhood experiences on adolescents' mental health (Stage 1). Collaboratively, priorities (touch points) for organisational responses will be identified (stage 2), and a low-burden resource will be co-designed (stage 3) and offered for implementation (stage 4) and evaluation (stage 5) in diverse settings. The study will provide insights into what adolescents and public sector organisations in the UK

want from a TIA resource, the experience of services/ organisations in implementing this and recommendations for resource development and implementation.

Ethics and dissemination The UK National Health Service Health Research Authority approved this study(23/WM/0105). Learning will be shared across study participants in a workshop at the end of the study. Knowledge products will include a website detailing the created resource and a youth-created film documenting the study process, the elements of the co-designed resource, and experiences of implementation. Dissemination will target academic, healthcare, education, social care, third sector and local government settings via knowledge exchange events, social media, accessible briefings, conference presentations and publications.

Strengths and Limitations

- This is a multi-site study nested study in a larger national study drawing on youth lived experience and survey data to inform the co-design of a resource to support diverse public sector settings to work in trauma-informed ways.
- We plan to recruit diverse young people to work in partnership with the organisations through which they are recruited, so that youth voice informs the co-design process. Our study also aims to understand how to deliver co-production studies with young people in ways that are trauma-informed.
- Use of frameworks for experience-based co-design and implementation theory is a strength.
- Implementation plans and evaluation protocols will be co-designed with settings, improving the chances of acceptable and meaningful resource use and evaluation.
- A limitation is that the time for implementation is relatively short, and not formalised, relying on participants adopting the interventions in their workplaces and evaluation stage is a simple real world pre-post assessment, not a trial.

Introduction

Adverse childhood experiences (ACEs) include events such as verbal, sexual, or physical abuse; neglect; parental separation or incarceration; substance misuse, domestic violence, or family mental illness, bullying, poverty, racism, death of significant others and multiple

For peer review only - http://bmjopen.bmj.com/site/about/guidelines.xhtml

losses(1, 2). Children who have experienced ACEs are at elevated risk of poor mental health outcomes compared to children who have not experienced one of these ACE(3). Experiencing multiple ACEs can have particularly detrimental effects on a physical, mental, social and economic outcomes in childhood, adolescence and adulthood.(4, 5)

Many ACEs meet the definition of trauma(6) as events that involve actual or threatened death, serious injury or sexual violence either by witnessing it or hearing about it.(7) Although many ACEs (e.g. homelessness) are not included in this definition of trauma, many ACEs have trauma effects (1). In our work, ACEs are considered as potentially traumatising, especially in the absence of buffering, supportive relationships and contexts. Our focus is the long-term impact of ACEs on later adolescent mental health.

Trauma can powerfully shape a young person's psychobiology(8), influencing their mood, thoughts, emotions and behaviours in adolescence as well as how they view, make sense of and react to events and people (e.g. being on alert for harm from people, anticipating negative outcomes). Such responses are adaptive following trauma, and dynamic in that responses to new traumas may change. However, usually the style of adaptation tend to persist even when a trauma has stopped. The response can overgeneralise into non-trauma situations (Lis et al., 2020) as threat perceptions are altered and sensitised.

Adaptive responses can include avoiding specific reminders, loud noises, too many people, or the need to calm and self-soothe at a time of anxiety and autonomic arousal linked to the flight-fight response. Alongside these there may be emotional dysregulation and impulsivity and inability to plan if a person is affected by flashbacks and distress, as if the original event is being repeated. Therefore, a person's 'adaptive' behaviour and their responses can easily be misunderstood or misrepresented by others as unhelpful, disproportionate, confusing or defensive, or even showing poor conduct and unprofessional. This may result in actions by the teacher or employer, for example, to apply rules and codes of conduct, and sanctions.

These can then be felt as a form of re-traumatisation; this occurs when experiences that are mundane to others (e.g. meeting professionals, being asked to consent to a process) remind the person of their past trauma, and a trauma-based response is evoked (e.g. submission, disengagement, fear-based aggression)(9). Many community organisations and services, from schools to youth organisations and healthcare, routinely encounter young people who

For peer review only - http://bmjopen.bmj.com/site/about/guidelines.xhtml

may have experienced ACEs but may be unaware of how the young person's trauma may be shaping their needs, engagement and behaviour with them(10, 11).

Since the early 2000s, there has been increasing global attention to the ways that organisations, services, institutions and settings (hereafter organisations) could work in trauma-informed ways (12, 13). Trauma-informed approaches (TIA) aim to improve the capacity of professionals and services to respond helpfully to people who may have experienced trauma. TIAs, although still being defined, concur that the aim is not to treat trauma-related difficulties, which is the remit of trauma specific services. Rather, TIAs focus on people and processes, and seek to improve organisational understanding that trauma is often inflicted by someone previously trusted by a young person, and to appreciate the prevalence and impact of trauma on a person's neurological, biological, psychological and social development, as well as how surviving adversity may be shaping a person's coping strategies(14),(15). TIAs align with a social model of disability, which views societal lack of adaptation and flexibility to be generating disability rather it being an innate property of the person. The key principles of a TIA, which have broad international recognition, are safety, trustworthiness, choice, collaboration and empowerment(12, 16). Yatchmenoff et al. (14) summarise these as safety, empowerment, and self-worth.

In the UK, the government has produced guidance on TIAs for the health and social care sectors(17). The guidance presents TIAs as understanding how the trauma-imprint can affect a person's ability to develop trusting relationships with organisations and staff alongside the importance of minimising the risk of re-traumatisation (which can undermine recovery) and of increasing a sense of control and autonomy for the trauma survivor.(12) The way organisations function, their policies and procedures, and how they engage with and respond to people can all carry re-traumatisation risks(18). The guidance also acknowledges that trauma, both direct and vicarious, can affect staff and thus protecting their wellbeing is also a feature of a TIA(16).

TIAs are widely recommended, and often implemented, across public sectors in the U.K (16, 19, 20) including healthcare, education and social services. Although the evidence of effectiveness is only emergent, and is highly complex to gather, TIAs appear to have potential to be beneficial for ACE-affected populations(21, 22) (23). However, despite an

For peer review only

For peer review only - http://bmjopen.bmj.com/site/about/guidelines.xhtml

organisation's commitment to ideology of a TIA, they can, like any complex intervention, be hard to implement and sustain (24, 25). They require change at a systemic level to support paradigm shifts across policy, procedures and practices, finding ways to in trauma-informed ways in every interaction and to prioritise the building of trusting, mutual relationships(26). Organisations need support to become TI.

Multiple resources and toolkits have been developed internationally to assist organisations in implementing a TIA(27, 28). Toolkits have begun to emerge in the UK. For example, the Roots framework(26) is a self-assessment tool facilitating organisational reflection on current and possible practice, including in National Health Service settings. The Scottish government have produced a Trauma-Informed Practice Toolkit for sector-wide organisations to self-evaluate and plan action across ten implementation domains(29). However, despite a proliferation of resources for TIAs, young people's perspectives in shaping these remain rare. There is also a need for continued learning on successful implementation of TIAs, to see what TIAs look like in practice in different human services organisations, the barriers encountered and how these can be overcome(14). A further significant gap in knowledge is the impact of TIAs. Existing evidence comes mostly from clinical settings, where professionals are likely to have specialist trauma training. There has been little evaluation of TIAs in UK public sector organisations whose primary focus is not clinical care.

Our *Attuned to Trauma* study aims to contribute to filling these knowledge gaps. We present our protocol for this study which is nested within a large, UK project (*Attune*) examining ACEs and pathways to mental health outcomes in adolescents. Our nested study builds on the learning from two early work streams in Attune, spanning arts-based lived experience and data set analyses. Combining insights from those work streams and using accelerated experience-based co-design (AEBCD) principles(30), we bring young people and public sector organisations together to co-design, implement and evaluate a new youth-informed resource to support TIA in organisations relevant to adolescents (10-24y) in the UK. We utilise this age range as it is encouraged by the project funders (UK Research and Innovation) and reflects calls to appreciate the earlier onset and extension of this life phase, arguably driven by social, digital, marketing, education and economic forces (Sawyer et al., 2018). Working with young people, via participatory and co-design research methods, can

For peer review only - http://bmjopen.bmj.com/site/about/guidelines.xhtml

increase the chances of service improvements which are sensitised to their needs and lived experience, and therefore more likely to be effective. Our project aligns with co-production theory that 'service users' should not be seen as passive recipients of that service but as active citizens with something of value to contribute. Treating young people's perspectives as an equitable form of knowledge is a core theme of the UKRI funding stream for this project. We therefore bring professionals and young people into dialogue and co-creation together.

Study Aim

This study aims to co-design, implement and evaluate a public health resource to help diverse public sector organisations in the UK to work in TI ways with adolescents and young people (10 - 24y). Our study also aims to contribute to understanding about how to deliver co-production studies with young people in ways that are trauma-sensitive.

Research questions

We address two primary research questions:

1. What do young people and stakeholders want in a co-designed UK resource to improve public sector capacity to work in trauma-informed ways?
2. Is such a co-designed resource acceptable, feasible, efficacious and affordable, and how should it be refined for future implementation in the UK public sector?

2.0 Methods

2.2 Design

This is a co-design and pre-post evaluation study nested within the UK Attune project. The co-design stage draws on Experience Based Co-Design (EBCD) which is widely used to design health service improvements. Central to EBCD is an initial discovery phase, which brings together services users and service providers in a balanced power relationship to discuss the personal experiences of service users and key moments in the service that shaped this. Services users and providers are then supported to co-design service changes to improve the quality of care(31). We will adopt an accelerated form of EBCD, termed AEBCD, which can be effective in achieving similar aims to standard EBCD. AEBCD typically brings pre-

For peer review only - http://bmjopen.bmj.com/site/about/guidelines.xhtml

existing narratives of experiences from relevant groups to start the co-design process, establishing where the co-design group identify consensus or divergence from those narratives. We will do this by bringing early Attune insights from adolescents (instead of the usual service user interviews), and adolescent-produced art, which includes film, and narratives to convey lived experience (instead of a purposefully commissioned service user 'trigger' film). We retain the core principle of EBCD in that the experiences of young people will drive co-design of organisational resource(s) for a TIA. The produced resource(s) will be shared with specific organisations to explore how they chose to implement it. We will conduct a preliminary evaluation of this.

Normalisation Process Theory (NPT) and principles of diffusion of innovation(32) in service organisations will inform our approach to resource co-production, trial implementation and evaluation, acknowledging we are at the earliest stage of innovation for service development. NPT characterises implementation as a social process of collective action. It proposes concentration on four domains at all stages of complex intervention design, delivery and evaluation, namely; intervention coherence, cognitive participation (engagement), collective action (required to enable the intervention to happen) and reflexive monitoring (of the costs and benefits of the innovation). Attention to these domains will structure our project approach. We will supplement these with the most relevant domains influencing the diffusion of innovations in services and organisations, e.g., characteristics of the system, the innovation and anticipated individual adopters. (32, 33)

2.3 Setting

This is a multi-site study taking place across three regions of England, namely Cornwall, Kent and West Yorkshire These sites are chosen to support participation by young people and stakeholders from rural, coastal and urban regions. Place is important in understanding ACEs (34, 35) (36) and in understanding local organisational cultures and resources for change.

2.4 Participants

2.4.1 AEBCD stage

There is little literature to inform trauma-informed, co-design methods (see (37, 38)for example in primary healthcare intervention co-design), although principles of co-design

For peer review only - http://bmjopen.bmj.com/site/about/guidelines.xhtml

overlap with some principles of a TIA (e.g. collaboration, choice). We aim to contribute to emerging discussions about how to deliver trauma-informed co-production methods by recruiting five young people from the Attune regional young people's advisory groups (YPAGs) to form a working party to design the AEBCD workshops to be trauma-sensitive. The YPAGs are based in several regions in the UK: Cornwall, Kent, London, Oxfordshire and Yorkshire. The YPAGs comprise young people between 10-24y with diverse identities (e.g. in terms of neurodivergence, gender identity, ethnicity and gender).

Sample sizes for EBCD studies vary. The recommended number of services users is between 5-15. For our AEBCD workshops, we will aim to recruit 5-8 young people (10-24y) and between 5-8 professionals to attend workshops in each of our UK regions (Cornwall, West Yorkshire and Kent; highest total n = 48). Co-design processes need to be responsible to participants with whom power is shared; therefore, despite this intended plan, we will retain flexibility to respond to the needs of young people and stakeholders who may prefer session in smaller or larger groups, or in a modified format.

Professionals will be recruited from diverse public sector organisations in our nominated regions. This may be through the Attune project network or by first approaches to local councils, local authorities, and / or third sector organisations. We will seek organisations who have a helping role with young people, e.g. schools, local authorities, colleges and universities, youth offending teams, community support organisations, housing associations etc. Bringing diverse organisations together to work on TIAs can be effective when they are supported to focus on their similarities, common challenges and collective solutions rather than differences(14). Working in local, regional contexts can support interprofessional learning(39). To be eligible, organisations must confirm they are ready and motivated towards TI ways of working and commit to sending up to two organisational members of staff to all AEBCD workshops and to supporting the recruitment of two to three young people from their setting with whom they can work in partnership.

Young people will be recruited via their participating organisation / setting. Participating organisations will be asked to identify 2-3 young people from their setting who are: between 10-24y; able to give consent and, if under 16, provide guardian consent; have experienced trauma and / or ACEs; and able to contribute to AEBCD workshops (conducted in English) where ACEs and trauma will be discussed. That recruited young people will have

For peer review only - http://bmjopen.bmj.com/site/about/guidelines.xhtml

experienced trauma and / or ACEs will be determined via three means: (i) knowledge that the recruiting setting has about the young person whom they deem suitable to approach for study participation; (ii) our recruitment information to young people will include experience of trauma / ACEs as an eligibility criterion; and (iii) via a survey administered as part of the larger Attune survey that includes items about experiences of ACEs and trauma. In considering potential participants, organisations will be asked to consider approaching underrepresented groups where possible (i.e. young people from minority communities, with diverse identities in terms of place, gender, ethnicity, LGBTG+ and neurodivergence).

Organisations will be asked to approach potential participants with study information and explore their interest in taking part and to secure informed consent from them and their guardians as needed. All young people will be paid for their time in line with national guidance(40).Requiring guardian consent for under 16s, which is mandated by the approving ethics committee, may prohibit some young people from participating (e.g. those still living in adverse environments). Participation by professionals and young people is opt-in.

Patient and public involvement

Patients and/or the public were involved in the design, or conduct, or reporting, or dissemination plans of this research.

2.4.2 Implementation and Evaluation

All organisations who participated in the AEBCD across our study regions will be eligible for the next stage of implementing and evaluating the co-designed resource for 6 months.

2.5 Procedure

2.5.1 AEBCD stage

Pre-Workshop Preparation (Summer 2023)

Taking part in co-production for TIAs may generate individual and organisational concerns, e.g. around one’s own trauma, managing personal and organisational disclosures, or about organisational limits for change. As part of our efforts to design trauma-informed co-

For peer review only - http://bmjopen.bmj.com/site/about/guidelines.xhtml

production methods, we will prioritise the fostering of psychological safety, a first principle of a TIA, for organisation staff. Applied to working practices, psychological safety is seen as essential to 'unfreezing' organisations so they can learn and change (41) and is manifested by "the willing contribution of ideas and actions to a shared enterprise" ((42) p. 24). It can be fostered by reducing perceived threats and interpersonal risk and encouraging provisional tries towards change (43). Therefore, using a topic guide informed by NPT and Greenhalgh et al.'s (32, 33) work on developing and implementing complex interventions, we will conduct approximately three individual online pre-workshop orientation meetings with organisations to help us understand their reasons for taking part, their needs and expectations of the study, the main demographic of young people they encounter, their current status with regards to TIAs, any strategic objectives informing their current or planned organisational delivery or organisational practice, their perceived risks around participation and how we can mitigate these (i.e. system antecedents and readiness for innovation).

We will also endeavour to promote psychological safety for adolescent attendees. For adolescents, psychological safety can means feeling able to take interpersonal risks because there is little fear that this will result in embarrassment, ridicule, or shame and so it enables people to engage, connect, change, and learn(42). Positive relationships, allyship through working with peers similar to you, and control over contributions can all build psychologically safe working contexts for adolescents(44). Towards this, we will conduct online engagement meetings with adolescents to begin to build positive relationships, to understand their motivations, needs, expectations and concerns about participation, and how we can mitigate any perceived risk, and optimise their ability to contribute. Adolescent attendees will also be invited to meet each other online before the first workshop and to identify shared ambitions for their involvement.

With consent, and with the help of the youth working party, appropriate information from the online pre-meetings with organisations and young people (e.g. strategic objectives of the organisation, perspectives on how to contribute) will be synthesised into a collaborator's booklet so that organisations and young people are aware in advance of who they will be working with in the workshops and how people can contribute and benefit differently.

For peer review only - http://bmjopen.bmj.com/site/about/guidelines.xhtml

Signed informed consent for the ECBD workshops will be secured after these pre-meetings from all confirmed attendees. Our learning from these meetings will shape workshop design. We will also secure opt-in or opt-out preference for photography and filming in the workshops.

AEBCD Workshop Series

We will conduct four full day AEBCD workshops over 18 months; three before the evaluation stage and one after for optimisation and lessons learned from the evaluation. Table 1 shows the aims and anticipated outcomes of each workshop and Figure 1 shows the study timeline and connection with the main Attune project. Young people will lead many workshop sessions and workshop facilitators will be members of the Attune research team who will be trained and follow a standardised format for each workshop.

(FIGURE 1 the timeline of the study and connection to wider Attune Project)

Workshops will involve activities and discussion in small and large groups. Discussion questions will be shaped by the youth working party so they are framed in ways that are meaningful and accessible to adolescent attendees; as far as possible, planned questions and activities will be sent to all attendees in advance of workshops. Conventions in EBCD are to use basic but effective means of securing participants' engagement (e.g. creative activities, scenarios, case discussion) and responses (e.g. flip charts, post-it notes, maps. Sketches etc). We offer examples below of the types of activities we may use and additional detail is given in Supplementary Materials. We will emphasise our valuing of multiple ways of contributing beyond talking (including listening, responding to anonymised digitised questions such as Mentimeter©, post-event feedback) from which we can learn. These are features of psychologically safe ways of working with young people. We will seek brief workshop evaluation via email after each workshop.

Data collection

For peer review only - http://bmjopen.bmj.com/site/about/guidelines.xhtml

AEBCD workshops will be audio-recorded (small group and/or whole group level) and field notes will be taken by trained facilitators. We will collect all workshop artefacts (e.g. creative outputs, written and digital responses) to support analysis. As soon as possible after each workshop, facilitators will meet to share notes and to reach consensus on key insights from the events. -At the end of the AEBCD stage, we will evaluate our approach using the Public and Patient Engagement Evaluation Tool which assesses the quality and impact of public and patient engagement activities within health system organisations(45).

Data analysis

The key moments in the workshop audio-recordings will be transcribed (i.e. those which are pertinent to learning, excluding for example, team building exercises). Transcribed extracts will only be linked to organisation type when essential, e.g. when knowledge of the setting type is required to comprehend the point / discussion. Full anonymisation of any other identifying details will occur at the point of transcription. Analysis will be conducted at regional levels before being merged for whole group analysis. We will merge the 'key insights' field notes with workshop artefacts and 'key moment' in the audio recording, initially framed in terms of workshop objectives (e.g. to understand views on an issue or to identify priorities). We will share emerging outcomes with the wider Attune project for credibility and sense-checking at key points. Lay summaries of each workshop will also be shared with participants prior to the next workshop, where they will be invited to approve or improve our recording and interpretation of learning from the preceding workshop. Data storage and sharing will be in line with the data management plan for the Attune project.

Workshop 1 (Autumn 2023)

The EBCD discovery phase typically involves understanding the organisation and 'service users' lived experiences. Workshop 1 is a discovery phase where we share Attune Workstream 1 data on the lived experiences of ACE-affected young people, generated using arts-based approaches, their views on how this has affected their mental health and how the insights are relevant for organisations. We will also present insights from Attune Workstream 2 which involves the analysis of several existing, large UK datasets to identify determinants of adolescent mental health risk and resilience following ACEs. Discussion will

For peer review only - http://bmjopen.bmj.com/site/about/guidelines.xhtml

then explore key touchpoints (46)namely (i) how these insights about the imprint of trauma on adolescents lead organisations to reflect on and identify some prominent current practices which may be unattuned to this knowledge; and (ii) what kinds of help organisations feel would help them to become more youth- and trauma-informed. We avoid introducing existing tools at this point to ensure service and service-user led solution.

Workshop 1 → Workshop 2

In line with our data analysis plan, we will examine how the discovery phase impacted different sectors / organisations and where they identified priorities and opportunities for change. Our analysis will be attentive to organisation type if necessary (i.e. if differences emerge). Outcomes will be summarised by region first and then combined with outcomes from the other study regions, where appropriate. Regional and merged outcomes will be shared with regional attendees who will be encouraged to share and discuss this in their organisation.

Workshop 2 (Spring 2024)

As stimuli for Workshop 2, and if Workshop 1 data indicates relevance and need, we will share in advance with participants two existing UK toolkits for organisations to become more trauma-informed(26). These toolkits are recent and evolving but are not yet youth-informed and are resource-intensive to implement. In Workshop 2, we will begin co-design of a low-intensity, youth-informed organisational resource. The aims of this workshop are to (i) identify modifiable ways of working to become trauma-informed and which are highly likely to be of benefit to adolescents; (ii) likely to be adopted (as per Greenhalgh et al., 2004 characteristics of the innovation and system); and (iii) begin to develop the nature of resource to be created. If consensus from co-design is lacking across our regional sites, we will convene a small working group with representation from each site to produce a final set of priorities (47) . These will especially focus on specifications that permit the resources to be adopted for particular sectors, regions, and intersectional characteristics encountered in stakeholder organisations.

For peer review only - http://bmjopen.bmj.com/site/about/guidelines.xhtml

For peer review only

Workshop 2 → Workshop 3

Once outputs are analysed, the research team and youth working party will create up to four outline prototype resources that satisfy some of the needs expressed in the workshops. We will also produce draft implementation and evaluation recommendations, all shared in advance of Workshop 2, so that organisations have opportunities to discuss at a local level.

Workshop 3 (Easter 2024)

The aim of this workshop is to select and build the core components of a final resource, as well as final recommendations for its implementation and evaluation. Workshop outputs will inform development, with the youth working party, of a final resource ready for the implementation and evaluation phase.

Workshop 4 (Spring 2025)

After the implementation and evaluation phase, Workshop 4 will bring participants back together to (i) share group-level analysis of outcomes and experience of implementation and (ii) key aspects of the resource to refine for testing in future studies. We will capture organisational plans to continue with any TIA changes, as well as youth and professional evaluations of study participation, including psychological safety.

For peer review only - http://bmjopen.bmj.com/site/about/guidelines.xhtml

Table 1: Aims and anticipated outcomes from AEBCD workshops and informing concepts

Workshop	Primary Aim	Secondary Aim	Outcomes	Informing Concepts
**1**	To share lived experience and data insights from and about young people's ACEs and trauma (from Attune) to prompt reflection on organisational change.	Establish collaboration, model a trauma-informed way of working, promote youth voice, understand the readiness and needs of organisations from youth and professional perspectives to act on new knowledge.	Learning what the data means to diverse sectors and where organisations recognise need, motivation and opportunity to operationalise a TIA.	Linkage*<br>Communication & Influence*<br>Absorptive capacity for new knowledge*<br>System readiness*
**Methods**	Presenting Attune art-works, films, key themes from Attune WS1 (lived experience extracts) and WS2 (outcomes of national dataset analyses). Enquiry activities about participants' views of this, the level of resonance / divergence, and what it directs attention to for public sector settings.	Pre-workshops communications and meetings to understand and build readiness and collaborative intention; being clear about the day's structure, content and purpose; dedicating safe spaces in the workshop and a trauma-informed facilitator to support young people; careful orientation (ice-breakers) opportunities; workshop activities based on play and arts (suitable for all ages) and multiple ways of contributing (drawing, writing, hand signals).		
**After Workshop 1:** synthesise outcomes across regions ready for sharing at Workshop 2.<br>If appropriate, share existing organisational TI principles and toolkits as stimulus.				
**2**	To identify priority, modifiable ways of working to become trauma-informed and identify the nature of a resource to be created.	To strengthen partnerships, to understand where youth and organisational perspectives on the nature of a TIA align and where they do not; and to understand the values and motivations of organisations for change, and what they need from a resource to	Learning what organisations need from a resource, the most modifiable aspects of their ways of working, and likely benefit to them and young people.	Linkage*<br>The innovation*<br>Adopter*<br>System readiness*

For peer review only - http://bmjopen.bmj.com/site/about/guidelines.xhtml

		help them do this, some expected outcomes for evaluation		
**Methods**	Exploration of a select number of existing tools; imagination tasks for co-design; consensus-reaching activities (nominal group technique; bullseye tasks); co-design activities	Invigorating (through words) project ambition and potential through partnership; relationship building activities; activities exploring differences and similarities in adolescent and professionals' priorities; play building activities to explore opportunities and challenges in co-design		
**After Workshop 2:** synthesise outcomes and share. Produce toolkit prototypes, and draft implementation and evaluation plans				
**3**	To select and refine core components of the final resource prototype	Decide on implementation options for the next phase and establish priorities for the evaluation.	Prototype resource and recommendations for implementation and evaluation.	Linkage* Implementation Process*
**Methods**	Goal agreement; Nominal group technique; priority rating activities for outcomes	Activities to explore aspects of NPT with professionals and (separately) to explore pro and cons of implementation options with young people; implementation roadmap design		
**After Workshop 3:** Refine prototype resource, implementation plan and evaluation protocol.				
**4**	To review findings from the implementation and evaluation phase and improvements needed to the resource	To capture implementation experiences and recommendations for an improved implementation protocol; to understand lessons learned and ambitions of youth and organisation for TIA in the future	Understanding how to improve the resource, and its implementation and evaluation, ready for future testing.	NPT domains of evaluation: coherence, cognitive participation, collective action, reflexive monitoring**
**Methods**	Arts-based activities to capture experience; case discussions; comparative outcomes to expectations	Priority rankings for learning and development; creating key messages banners		

For peer review only - http://bmjopen.bmj.com/site/about/guidelines.xhtml

* From Greenhalgh et al (2004) conceptual model of the determinants of diffusion, dissemination and implementation of innovations in health service delivery and organisation.

**NPT (Murray et al., 2014)

For peer review only

For peer review only - http://bmjopen.bmj.com/site/about/guidelines.xhtml

2.5.2 Implementation and Evaluation

Implementation of new innovations can be optimised by the involvement stakeholders in the earliest stages of design and ownership over local and flexible implementation(32, 48). Organisations involved in our AEBCD stage will be invited to implement the resource in their setting for a minimum of six months. Although AEBCD will produce implementation recommendations, organisations will have autonomy in adapting the resource and its implementation to their setting, reflecting a supported 'let it happen' approach to learn from organisational agency and creativity in implementation(49). This is in line with moves away from prioritising intervention fidelity over local tailoring, especially in the learning phase of interventions (50). As a minimum, and in line with recommendations for TIA, organisations will be encouraged to establish an implementation team to lead and champion resource implementation in their setting (14).

Evaluation of the impact of TIA on service users is extremely complex (23) and is not the focus of our early-stage evaluation. We will evaluate resource implementation. Administering a standardised pre-post measure of an organisation's TIA may not be feasible as there are no UK generated measures suitable for evaluation of TIA in diverse settings such as those likely to be involved in our study. Thirkle et al.'s review (51) of measures of TIAs shows that most tools originated in the United States and are not easily tailorable beyond healthcare organisations(12). Decisions on whether to utilise a standardised measure or to generate a bespoke pre-post evaluation tool will be make in the AEBCD stage. Evaluation will be informed by NPT and relevant domains of diffusion of innovations in services (32, 33, 48). It will include, as a minimum, a survey of how organisations deployed the resource and audio-recorded, key informant semi-structured interviews, following a topic guide, to explore: the experiences of organisations; barriers and solutions to resource use; any unanticipated benefits or adverse impacts; lessons learned and recommendation for improvements, testing and upscaling. We will also consider the extent to which the NPT parameters for optimal implementation were met: (coherence, cognitive participation, collective action and reflexive monitoring). As in all intervention research, it is important to learn if outcomes, especially poor ones, were influenced by the resource itself or by challenges in implementation (52). Qualitative data will be analysed using Framework Analysis, which is suitable for mixed methods data in health research(53, 54).

For peer review only - http://bmjopen.bmj.com/site/about/guidelines.xhtml

Across the review period for this protocol, a small number of changes were made to our methods, namely: (i) for clarity, naming our process as AEBCD rather than EBCD; (ii) reducing our AEBCD sample size from n=15 young people and n=15 professionals in each region to n=5/8 of each per region to optimise the experience of diverse young people in the workshops; (iii) dropping purposive sampling in the implementation phase so that more settings will be eligible to test the resource; and (iv) removing the plan to conduct a system readiness assessment of settings prior to implementation in order to reduce the burden on participating settings.

3.0 Ethics and Dissemination

This study has received ethical approval from the UK National Health Service Health Research Authority (23/WM/0105 June 2023). Recruitment for the project will commence in summer 2023 and all study activity will be completed by August 2025. Organisations and participants will receive age-appropriate verbal and written information about the study, including as much transparency as possible about the workshops, who will be there, what is involved and how we are endeavouring to make the workshops psychologically safe. Participants will be reminded that they are free to take breaks in the workshop, to contribute in ways that are right for them, including just listening. Support for participants will be available in the workshops from trained clinicians and options for support beyond this will be made clear. Organisational consent for the implementation phase will be secured after the AEBCD stage. For any level of study participation, we will secure signed informed consent, with additional guardian consent for under 16s, and opt-out consent for the filmmaking components. All participating adolescents and organisations, and their data, will be fully anonymised in any study outputs.

Dissemination will be within this study first, via Workshop 4 in the AEBCD series to the study participants and organisations, and then to the larger Attune study. Wider knowledge dissemination will be in partnerships with adolescents and participating organisations, and will target academic, healthcare, education, social care, third sector and local government settings via knowledge exchange events, social media, executive reports, conference presentations and publications. We will share learning and insights about (i) conducting AEBCD on trauma projects with adolescents and UK public sector settings, using arts-based

For peer review only - http://bmjopen.bmj.com/site/about/guidelines.xhtml

approaches on lived experience; (ii) the concerns, priorities and opportunities adolescents and different sectors perceive for working in more trauma-informed ways; (iii) the resource they produced and how it was implemented in diverse settings; (iv) how resources for TIAs in diverse settings could be evaluated; and (v) next steps for resource refinement and upscaling, if appropriate. Knowledge products will include a study website detailing the AEBCD process, the resource, evaluation and outcomes, and a youth-created film documenting the study process and organisational experiences of implementation.

Discussion

Trauma is so widespread and its effects so significant that is it has been positioned as a significant public health issue. ACEs and trauma are highly predictive of poor outcomes in all areas of life and across lifespans, with adolescence being a time of particular vulnerability to the presentation of poor mental health in ACE-affected young people. Trauma-informed ways of working with young people strive to understand the ways that their ACEs may be shaping their needs and engagement with a service, with efforts to minimise re-traumatisation. Although resources are emerging in the UK to support public sector settings to be trauma-informed, we are still at a preliminary stage of understanding about how they may be best implemented and sustained and whether they meet the needs of young people.

To our knowledge, this is the first AEBCD study working across different UK public sector settings to co-design a resource that could help professionals be more attuned to the needs of adolescents in their settings who have lived through ACEs and / or trauma. Our study turns into action the learning from the arts-based participatory methods in the main Attune study which captured the lived experience of diverse, ACE-affected adolescents in multiple UK regions. A number of strengths benefit the study. Involvement of different public sector settings allows us to identify commonalities in needs. We support collaboration between adolescents and professionals, inviting them to join the project together and work in dialogue across the AEDCD workshops. We address the need to better understand how resources for TIA can be implemented and evaluated in the public sector, driven by settings / services themselves and informed by NPT. This study will produce new insights on what

For peer review only - http://bmjopen.bmj.com/site/about/guidelines.xhtml

adolescents and professionals want in a co-designed UK resource to improve their capacity to work in trauma-informed ways, and whether that resource is acceptable, feasible, efficacious. This contributes to the national agenda for developing trauma-informed approaches for young people.

The study has some limitations. Recruitment of young people under 16 will be subject to guardian consent; this may exclude young people who do not wish to or cannot secure that consent. The AEBCD process of three workshops may make project participation possible for stretched organisations who can only give limited time to research projects but means deep exploration and creativity may be compromised. Bringing diverse settings together may risk consensus-reaching on the type of resource to be created. The effects of trauma-informed organisational interventions can be slow to emerge and examine. Thus, in our time-limited project, our is an exploratory evaluation of their experiences implementing the resource to inform resource improvements, more effective implementation strategies and a more robust evaluation at a future point.

Funding:

This work was supported by the UKRI Medical Research Council. (MR/ W002183/1).

Patient and public involvement

Patients and/or the public were involved in the design, or conduct, or reporting, or dissemination plans of this research. Refer to the Methods section for further details.

Patient consent for publication

Not applicable

Competing interests:

None declared.

Acknowledgements

The authors wish to thank each member of the wider ATTUNE research team who have contributed to the wider work of the project.

For peer review only - http://bmjopen.bmj.com/site/about/guidelines.xhtml

Contributors

KB is the principal investigator for the ATTUNE study(ATTUNE — Department of Psychiatry (ox.ac.uk)) SHJ , KB and IB made substantial contributions to the conceptualisation and design of the study. IB and SHJ drafted the first version of the manuscript. IB, SHJ and KB reviewed the manuscript for intellectual content, approved the final version to be published and agreed to be accountable for all aspects of the work.

For peer review only

References

1. Varese F, Smeets F, Drukker M, Lieverse R, Lataster T, Viechtbauer W, et al. Childhood adversities increase the risk of psychosis: a meta-analysis of patient-control, prospective- and cross-sectional cohort studies. Schizophr Bull. 2012;38(4):661-71.
2. Felitti VJMD, Facp, Anda RFMD, Ms, Nordenberg DMD, Williamson DFMS, et al. Relationship of Childhood Abuse and Household Dysfunction to Many of the Leading Causes of Death in Adults: The Adverse Childhood Experiences (ACE) Study. American Journal of Preventive Medicine. 1998;14(4):245-58.
3. Norman RE, Byambaa M, De R, Butchart A, Scott J, Vos T. The long-term health consequences of child physical abuse, emotional abuse, and neglect: a systematic review and meta-analysis. PLoS medicine. 2012;9(11):e1001349.
4. Hughes K, Ford K, Bellis MA, Glendinning F, Harrison E, Passmore J. Health and financial costs of adverse childhood experiences in 28 European countries: a systematic review and meta-analysis. The Lancet Public Health. 2021;6(11):e848-e57.
5. Smith KE, Pollak SD. Early life stress and development: potential mechanisms for adverse outcomes. Journal of neurodevelopmental disorders. 2020;12:1-15.
6. Friedman MJ, Resick PA, Bryant RA, Strain J, Horowitz M, Spiegel D. Classification of trauma and stressor-related disorders in DSM-5. Depression and anxiety. 2011;28(9):737-49.
7. Association AP. Diagnostic and statistical manual of mental disorders (DSM-5®): American Psychiatric Pub; 2013.
8. Ridout KK, Khan M, Ridout SJ. Adverse childhood experiences run deep: toxic early life stress, telomeres, and mitochondrial DNA copy number, the biological markers of cumulative stress. Bioessays. 2018;40(9):1800077.
9. May CL, Wisco BE. Defining trauma: How level of exposure and proximity affect risk for posttraumatic stress disorder. Psychological trauma: theory, research, practice, and policy. 2016;8(2):233.
10. Luthar SS, Mendes SH. Trauma-informed schools: Supporting educators as they support the children. International Journal of School & Educational Psychology. 2020;8(2):147-57.
11. Spence R, Kagan L, Kljakovic M, Bifulco A. Understanding trauma in children and young people in the school setting. Educational and Child Psychology. 2021;38(1):87-98.

For peer review only - http://bmjopen.bmj.com/site/about/guidelines.xhtml

12. Harris M, Fallot RD. Envisioning a trauma-informed service system: a vital paradigm shift. New directions for mental health services. 2001;2001(89):3-22.
13. Maynard BR, Farina A, Dell NA, Kelly MS. Effects of trauma-informed approaches in schools: A systematic review. Campbell Systematic Reviews. 2019;15(1-2).
14. Yatchmenoff DK, Sundborg SA, Davis MA. Implementing trauma-informed care: Recommendations on the process. Advances in Social Work. 2017;18(1):167-85.
15. Fallot RD, Harris M. Creating cultures of trauma-informed care. Washington DC: Community Connections. 2009.
16. Fallot RD, Harris M. Trauma-informed approaches to systems of care. Trauma Psychology Newsletter. 2008;3(1):6-7.
17. Office for Health IaD. Working definition of trauma-informed practice. 2022.
18. Abuse S. SAMHSA's concept of trauma and guidance for a trauma-informed approach. 2014.
19. Long E. The future of pastoral care in schools: Exploring whole-school trauma-informed approaches. Pastoral Care in Education. 2022;40(3):342-51.
20. Mendel WE, Sperlich M, Fava NM. "Is there anything else you would like me to know?": Applying a trauma-informed approach to the administration of the adverse childhood experiences questionnaire. Journal of community psychology. 2021;49(5):1079-99.
21. Blanch A, Filson B, Penney D, Cave C. Engaging women in trauma-informed peer support: A guidebook. Alexandria, VA: National Center for Trauma-Informed Care. 2012.
22. Greenwald R, Siradas L, Schmitt TA, Reslan S, Fierle J, Sande B. Implementing trauma-informed treatment for youth in a residential facility: First-year outcomes. Residential Treatment for Children & Youth. 2012;29(2):141-53.
23. Department for Levelling Up HaC. Trauma-informed approaches to supporting people experiencing multiple disadvantage 2023.
24. Hodgdon HB, Kinniburgh K, Gabowitz D, Blaustein ME, Spinazzola J. Development and implementation of trauma-informed programming in youth residential treatment centers using the ARC framework. Journal of Family Violence. 2013;28:679-92.
25. Azeem MW, Aujla A, Rammerth M, Binsfeld G, Jones RB. Effectiveness of six core strategies based on trauma informed care in reducing seclusions and restraints at a child and adolescent psychiatric hospital. Journal of Child and Adolescent Psychiatric Nursing. 2011;24(1):11-5.
26. Thirkle SA, Kennedy A, Sice P, Patel P. The Co-production of the Roots Framework: A Reflective Framework for Mapping the Implementation Journey of Trauma-informed Care. medRxiv. 2022:2022.04. 13.22273691.
27. Henderson CM, Everett, M. Isobel S (Sydney LHD). Trauma-Informed Care and Practice Organisational

Toolkit (TICPOT): An Organisational Change Resource, Stage 2 - Supporting Organisational Change. Mental Health Coordinating Council (MHCC).
28. Addington D, Addington J, Schissel B. A depression rating scale for schizophrenics. Schizophrenia research. 1990;3(4):247-51.
29. Grandison AHaG. Trauma-Informed Practice: A toolkit for Scotland Scottish Government
30. The Point of Care Foundation : EBCD Experience based co design toolkit Point of Care Foundation
31. Moser A, Korstjens I. Series: Practical guidance to qualitative research. Part 5: Co-creative qualitative approaches for emerging themes in primary care research: Experience-based co-design, user-centred design and community-based participatory research. European Journal of General Practice. 2022;28(1):1-12.
32. Greenhalgh T, Robert G, Macfarlane F, Bate P, Kyriakidou O. Diffusion of innovations in service organizations: systematic review and recommendations. Milbank Q. 2004;82(4):581-629.
33. Greenhalgh T, Wherton J, Papoutsi C, Lynch J, Hughes G, A'Court C, et al. Beyond Adoption: A New Framework for Theorizing and Evaluating Nonadoption, Abandonment, and Challenges to the

For peer review only - http://bmjopen.bmj.com/site/about/guidelines.xhtml

Scale-Up, Spread, and Sustainability of Health and Care Technologies. J Med Internet Res. 2017;19(11):e367.
34. Crouch E, Radcliff E, Probst JC, Bennett KJ, McKinney SH. Rural-urban differences in adverse childhood experiences across a national sample of children. The Journal of Rural Health. 2020;36(1):55-64.
35. Wade Jr R, Cronholm PF, Fein JA, Forke CM, Davis MB, Harkins-Schwarz M, et al. Household and community-level adverse childhood experiences and adult health outcomes in a diverse urban population. Child abuse & neglect. 2016;52:135-45.
36. Whiteside-Mansell L, McKelvey L, Saccente J, Selig JP. Adverse childhood experiences of urban and rural preschool children in poverty. International journal of environmental research and public health. 2019;16(14):2623.
37. McGeown H, Potter L, Stone T, Swede J, Cramer H, group BG, et al. Trauma informed co-production: Collaborating and combining expertise to improve access to primary care with women with complex needs. medRxiv. 2023:2023.04. 21.23288783.
38. Cherry L. Trauma Informed co-production Guidance West Yorkshire Health and Care partnership.
39. Humphrey A, Eastwood L, Atkins H, Vainre M, Lea-Cox C. An exemplar of GP commissioning and child and adolescent mental health service partnership: Cambridge 1419 young people's service. Journal of Integrated Care. 2016;24(1):26-37.
40. NIHR. Payment guidance for researchers and professionals July 2022.
41. Schein EH, Bennis WG. Personal and organizational change through group methods: The laboratory approach: Wiley New York; 1965.
42. Edmondson AC, Lei Z. Psychological safety: The history, renaissance, and future of an interpersonal construct. Annu Rev Organ Psychol Organ Behav. 2014;1(1):23-43.
43. Frazier P, Richards D, Mooney J, Hofmann SG, Beidel D, Palmieri PA, Bonner C. Acceptability and proof of concept of internet-delivered treatment for depression, anxiety, and stress in university students: protocol for an open feasibility trial. Pilot and Feasibility Studies. 2016;2(1):28.
44. Wanless SB. The Role of Psychological Safety in Human Development. Research in Human Development. 2016;13(1):6-14.
45. Abelson J, Li K, Wilson G, Shields K, Schneider C, Boesveld S. Supporting quality public and patient engagement in health system organizations: development and usability testing of the P ublic and P atient E ngagement E valuation T ool. Health Expectations. 2016;19(4):817-27.
46. Donetto S, Tsianakas V, Robert G. Using Experience-based Co-design (EBCD) to improve the quality of healthcare: mapping where we are now and establishing future directions. London: King's College London. 2014:5-7.
47. Robert G. Participatory action research: using experience-based co-design to improve the quality of healthcare services. Understanding and Using Health Experiences–improving patient care. 2013:138-50.
48. Murray E, Treweek S, Pope C, MacFarlane A, Ballini L, Dowrick C, et al. Normalisation process theory: a framework for developing, evaluating and implementing complex interventions. BMC medicine. 2010;8:1-11.
49. May C. Towards a general theory of implementation. Implementation Science. 2013;8(1):18.
50. von Thiele Schwarz U, Hasson H, Lindfors P. Applying a fidelity framework to understand adaptations in an occupational health intervention. Work. 2015;51(2):195-203.
51. Thirkle S, Kennedy A, Sice P. Instruments for exploring trauma-informed care. Journal of Health and Human Services Administration. 2021.
52. Proctor E, Silmere H, Raghavan R, Hovmand P, Aarons G, Bunger A, et al. Outcomes for implementation research: conceptual distinctions, measurement challenges, and research agenda. Administration and policy in mental health and mental health services research. 2011;38:65-76.

For peer review only - http://bmjopen.bmj.com/site/about/guidelines.xhtml

53. Gale NK, Heath G, Cameron E, Rashid S, Redwood S. Using the framework method for the analysis of qualitative data in multi-disciplinary health research. BMC medical research methodology. 2013;13(1):1-8.
54. Rashidian A, Eccles MP, Russell I. Falling on stony ground? A qualitative study of implementation of clinical guidelines' prescribing recommendations in primary care. Health policy. 2008;85(2):148-61.

For peer review only

For peer review only - http://bmjopen.bmj.com/site/about/guidelines.xhtml

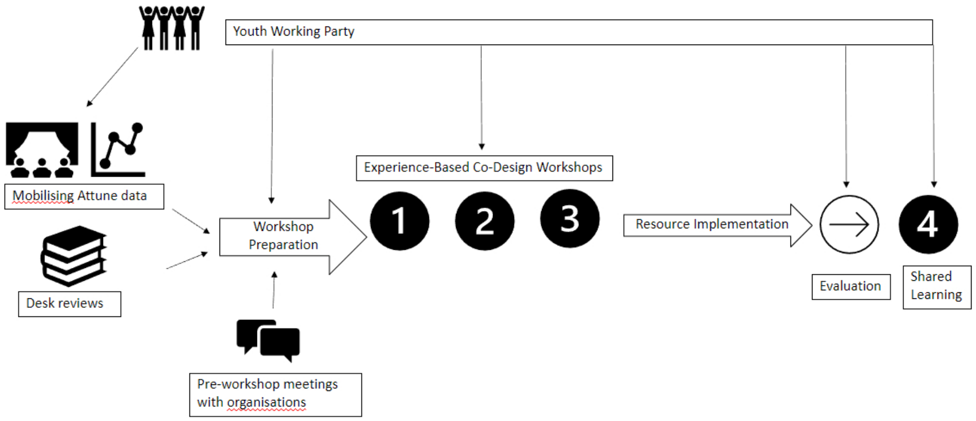

(FIGURE 1 the timeline of the study and connection to wider Attune Project)

307x157mm (96 x 96 DPI)

SUPPLEMENTARY MATERIALS

This table shows example activities that may be used in the AEBCD series in ways that can be tailored to different ages of young people, and which draw on creative practices and play.

	All ages	10-12 year olds	13-15 year olds	16+ year olds
Showing 'trigger films' (standard in EBCD)	Given context for films and purpose in workshop	Asked to spot 1-2 things in short clips (bingo). Given cards to show their view of important / not important / not sure in response to short clips.	Asked to write a word that captures what they felt was important in a clip. Collect and discuss as a group	Open discussion after clips. Encouraged to make brief notes during screening. Can use 'not sure' card
Sharing data / information	Presented in an engaging way; not using academic terms; giving examples; present as a quiz / myth busting / predicting what the information will say	Using thumbs up / down to indicate if they think it makes sense / is important / worth discussing.	Additionally asked for whether they are surprised by the data (thumbs up / down). Ideas for how we might explain the data / information to someone else.	In small groups, creating Venn diagrams / idea bubbles about what might they notice in the data / information and what might explain it
Persona / Character Play: designing a character with a back story to explore and better comprehend the nature of issues / diversity of experience	Given creative materials to design character in small group; asked to answer 'what has happened to your character'? 'how did that make them feel?' and 'what would they like to happen now'?	Supported to think about how movie characters are made up can still be about real life. Encouraged to focus on physical design of character first and then their story	Given guidance to think first about the big issue / experience they want their character to represent.	Encouraged to think about composite issues that a character could represent
Snowball Fight: people write an example on a piece of paper and in groups throw across the room for a set period. Pick up and read out examples near you at end.	Can write or draw the example, word they want to share.	Can ask someone else to read out word on the paper if wished.	Could be invited to think of the best word / example they can	Could be invited to add a brief statement word / example explaining why that is important to them
Forum Theatre: inviting or showing a live performance of a scenario, with pauses to ask the 'audience' different ways to respond / solve / change the scenario	Show example of how Forum Theatre works and how it can help creativity. Specific boundaries of response s as feasible and realistic in the real world.	Can be offered options if they find it difficult to imagine alternative responses and / or can focus ratings of big / medium / small problems in the scenarios	Supported to try one response option first; given time to consider impact and supported to report what they see.	Could hold opposing response options in mind and discussion in some detail
Bullseye Activity: refining the specific aims/ outcomes of a	Give examples of the pros and cons of specific vs.	Invited to design the bullseye page and work with same-age	Supported to think of 'wishes' or 'if only' as a	Supported to consider specific vs

For peer review only

resource or anticipated change	general aims, using everyday examples. Practising how we can vote for something as being near or far from our collectively determined bullseye	peers to generate ideas for outcomes, using comparisons to everyday examples	way to think about potential change / outcomes.	general aims via colour coding
Implementation mapping: decision trees / if-then steps	Shown examples of mapping resource implementation to understand choices and options, as well as importance of collective clarity	Use of simple ratings (Yes, No, Maybe) on cards for the most important choices. Moving these into piles to help see choices.	Use of 'must do', 'must not do', 'not sure' cards in response to options; adding of red and green flags for point of risk vs opportunity	Supported in groups to sketch starting point and key steps in implementatio n, considering what would be realistic in their setting.

For peer review only
